# Adverse weather conditions for UK wheat production under climate change

**DOI:** 10.1016/j.agrformet.2019.107862

**Published:** 2020-03-15

**Authors:** Caroline Harkness, Mikhail A. Semenov, Francisco Areal, Nimai Senapati, Miroslav Trnka, Jan Balek, Jacob Bishop

**Affiliations:** a School of Agriculture Policy and Development, University of Reading, Earley Gate, Reading RG6 6AH, United Kingdom; b Department of Plant Sciences, Rothamsted Research, West Common, Harpenden, Hertfordshire AL5 2JQ, United Kingdom; c Centre for Rural Economy, School of Natural and Environmental Sciences, Newcastle University, Agriculture Building, King's Road, Newcastle upon Tyne NE1 7RU, United Kingdom; d Global Change Research Institute (CzechGlobe), Academy of Sciences of the Czech Republic, Bělidla 986/4, 603 00 Brno, Czech Republic; e Mendel University in Brno, Zemědělská 1, Brno 613 00, Czech Republic; f Sustainable Agricultural Sciences, Rothamsted Research, West Common, Harpenden, Hertfordshire AL5 2JQ, United Kingdom

**Keywords:** Extreme events, Agroclimatic indicators, AgriClim, Sirius, CMIP5, Impact uncertainty

## Abstract

Winter wheat is an important crop in the UK, suited to the typical weather conditions in the current climate. In a changing climate the increased frequency and severity of adverse weather events, which are often localised, are considered a major threat to wheat production. In the present study we assessed a range of adverse weather conditions, which can significantly affect yield, under current and future climates based on adverse weather indices. We analysed changes in the frequency, magnitude and spatial patterns of 10 adverse weather indices, at 25 sites across the UK, using climate scenarios from the CMIP5 ensemble of global climate models (GCMs) and two greenhouse gas emissions (RCP4.5 and RCP8.5). The future UK climate is expected to remain favourable for wheat production, with most adverse weather indicators reducing in magnitude by the mid-21st century. Hotter and drier summers would improve sowing and harvesting conditions and reduce the risk of lodging. The probability of late frosts and heat stress during reproductive and grain filling periods would likely remain small in 2050. Wetter winter and spring could cause issues with waterlogging. The severity of drought stress during reproduction would generally be lower in 2050, however localised differences suggest it is important to examine drought at a small spatial scale. Prolonged water stress does not increase considerably in the UK, as may be expected in other parts of Europe. Climate projections based on the CMIP5 ensemble reveal considerable uncertainty in the magnitude of adverse weather conditions including waterlogging, drought and water stress. The variation in adverse weather conditions due to GCMs was generally greater than between emissions scenarios. Accordingly, CMIP5 ensembles should be used in the assessment of adverse weather conditions for crop production to indicate the full range of possible impacts, which a limited number of GCMs may not provide.

## Highlights

• Adverse weather conditions were analysed using a range of adverse weather indices.
• Future UK climate is expected to remain favourable for wheat production.
• Wetter winter and spring could, however, increase the risk of waterlogging.
• Use of global climate model ensembles to quantify prediction uncertainty is advised.

## 1 Introduction

Climate change is associated with a warming trend, as well as, increasing climatic variability and extremes (Rahmstorf and Coumou, 2011; IPCC et al., 2012; Kovats et al., 2014). Agricultural production is highly dependent on weather conditions, and extreme and adverse weather events beyond the normal conditions experienced by crops can have a dramatic impact on their yield. When coinciding with sensitive stages of crop development, adverse weather events including high temperature, late frost, heavy precipitation and drought can severely reduce crop yield and affect its quality (Deryng et al., 2014; Powell and Reinhard, 2015; Trnka et al., 2014). Severe cases of heat stress or prolonged drought can also lead to a total crop failure (Gourdji et al., 2013; Lesk et al., 2016; Trnka et al., 2014). The impact and increased frequency of adverse weather events may pose more of an immediate risk to food production, in comparison to changes in mean climate, since farmers have less time to adapt. Losses in agricultural production due to adverse weather conditions, alongside potential for high volatility in food prices, intensifies the challenge of ending world hunger and achieving food security by 2030 (target of the UN Sustainable Development Goals; Griggs et al., 2013), for a world population anticipated to increase to 9 billion by 2050 (FAO, 2009). As a result, adverse weather has been the focus of increasing attention in crop-climate modelling studies.

Wheat is the most widely grown cereal crop in the world (FAOSTAT, 2018; Lobell et al., 2012). As a temperate species the typical weather conditions of western Europe, including the UK, are favourable for wheat production (Reynolds et al., 2010). Approximately 40% (~1.8 million hectares) of the arable cropping area in the UK is dedicated to wheat production (DEFRA, 2018). Despite the relatively small acreage, the UK produces approximately 2% of the world's wheat benefitting from a high average yield of ~8 t ha^−1^, compared to a world average of ~3.5 t ha^−1^ (FAOSTAT, 2018).

Wheat is sensitive to various adverse weather conditions and abiotic stresses which can significantly reduce yields. Heat stress during anthesis can reduce grain number by affecting floret fertility (Alghabari et al., 2014; Mitchell et al., 1993; Porter and Gawith, 1999) and heat stress during grain filling can reduce grain size and quality (Nasehzadeh and Ellis, 2017; Savill et al., 2018). Late frosts, particularly those during ear emergence and early anthesis, can cause damage to the ear and yield loss (Al-Issawi et al., 2013; Fuller et al., 2007). Approximately 30% of wheat in the UK is estimated to be grown on drought-prone soils (Weightman et al., 2005). Prolonged water stress reduces leaf expansion and accelerates leaf senescence, and can reduce radiation use efficiency (Jamieson et al., 1998). Short-term drought episodes are also a particular issue for wheat at stem elongation and grain filling, causing a reduction in growth and crop-die back, while drought stress during reproductive development reduces grain number (Dong et al., 2017; Ma et al., 2017). Heavy rainfall prior to and at maturity can also lead to lodging and yield losses, as well as, a reduction in quality (Berry et al., 2003; Russell and Wilson, 1994). In addition, wet conditions during sowing and harvest can restrict farming activities and the ability to sow or harvest at the most appropriate time (Trnka et al., 2014, 2011).

Notable adverse weather events that have impacted wheat production in the UK include severe flooding in the summer of 2007, which was estimated to reduce cereal yields by approximately 40% in the flooded areas (Posthumus et al., 2009). Prolonged drought in 2011 affected growth of arable crops in England and Wales, followed by record high rainfall in the spring and summer of 2012, which in flooded areas reduced yields and delayed harvesting (Kendon et al., 2013; Parry et al., 2013). Prediction of the future occurrence of adverse weather events can, particularly at a large scale, be challenging due to the often localised nature of adverse weather events and uncertainty in future projections (Reyer et al., 2013; Seneviratne et al., 2012). Climate projections show a marked increase in summer heatwaves and heavy precipitation events for Europe (Kovats et al., 2014). There is considerable variability in projections across regions (Kovats et al., 2014; Powell and Reinhard, 2015), and seasons, with winters expected to become wetter and summers drier (Semenov, 2009). Previous studies predict the probability of adverse weather conditions for wheat may increase under a future climate, resulting in more frequent crop failure in Europe's key wheat growing regions (Trnka et al., 2014). Identifying areas within the UK which may be sensitive to particular adverse weather conditions is therefore an important area of research in understanding these climatic risks. Prior assessment of the adverse weather conditions which pose a risk to wheat production across the UK could aid in early decision making regarding choice of cultivars and crop management strategies. Previous evidence has either focused on a limited number of adverse weather events, for example heat or drought stress, or examined a range of events at a single station within the UK, which cannot identify spatial variations within the wheat growing area. This study examines the magnitude and spatial patterns of a range of adverse weather events based on different adverse weather indices across the UK, providing comprehensive analysis for adverse weather conditions which may pose a risk to wheat production under changing climate. The main objectives of the present study were to provide a comprehensive analysis of projected changes in the frequency, magnitude and spatial patterns of a range of adverse weather conditions for wheat production throughout the UK in the mid-21st century.

## 2 Methods

### 2.1 Study area

**Fig. 1:**
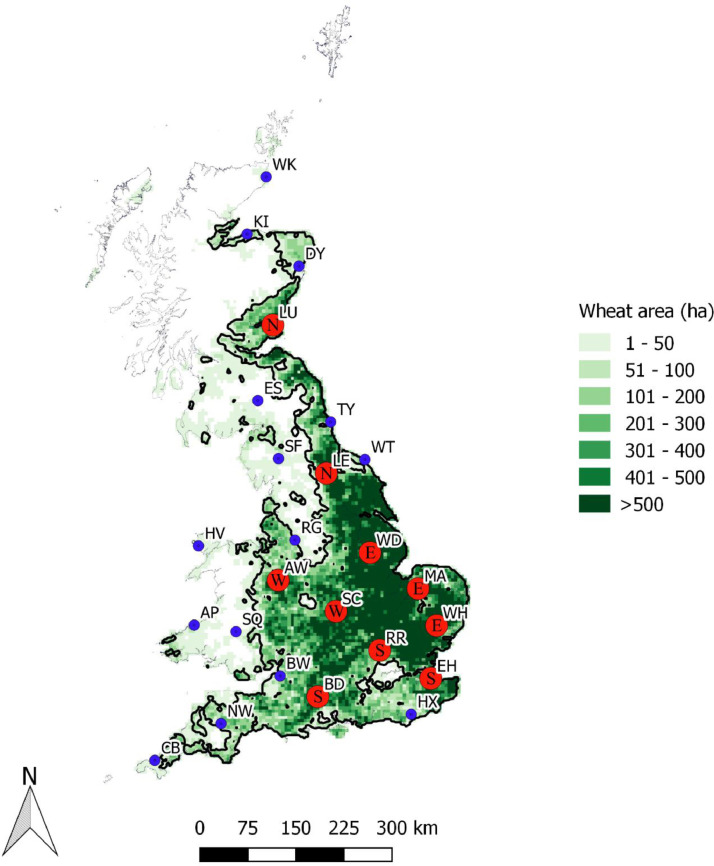
UK wheat cropped area 2010 (ha per 25 km^2^), data from EDINA (2018) including outline of key growing area. Location of the 25 UK sites included in the study (blue and red dots). Box plot results are presented for those sites with red dots (10 sites) and the letters within are used to split these 10 sites into 4 regions (referring to the cardinal direction of the site within the wheat growing area): north (N), east (E), south (S) and west (W). (For interpretation of the references to color in this figure legend, the reader is referred to the web version of this article.)

This study used daily weather data from 25 sites across the UK (Fig. 1). The 25 sites were selected from 85 climate stations within the Met Office network (Met Office, 2019) including sites which reported less than 10% missing values for temperature and precipitation, as well as, providing broad and even coverage of the key wheat growing areas in the UK.

### 2.2 Baseline and future climate scenarios

In the present study, the baseline climate was based on daily observed weather data during 1981–2010 including maximum and minimum air temperature, precipitation and sunshine hours (or solar radiation). We used quality control procedures (from Feng et al., 2004 and Durre et al., 2010) to identify and remove erroneous values which represented less than 0.1% of the dataset. To produce the local-scale future daily weather scenarios we used climate projections from 16 global climate models (GCMs; supplementary material) from the CMIP5 multi-model ensemble used in IPCC Assessment Report 5 (AR5) (IPCC, 2014). For the 2041–2060 and 2081–2100 climate scenarios (subsequently denoted as 2050 and 2090 respectively), two representative concentration pathways (RCPs) were used: a midrange mitigation scenario (RCP4.5) and a high emission scenario (RCP8.5). RCPs represent different targets of radiative forcing in 2100 i.e. 2.6, 4.5, 6.0, 8.5 W/m^2^ (van Vuuren et al., 2011). Corresponding CO_2_ concentrations (ppm) used in the simulations are presented in Table 1.

**Table 1:** CO_2_ concentrations (ppm) for the baseline, RCP4.5 and RCP8.5.

	Baseline	RCP4.5	RCP8.5
1981 - 2010	364		
2041 - 2060		487	541
2081 - 2100		533	844

### 2.3 Construction of the local-scale climate scenarios using LARS-WG

Due to the coarse spatial and temporal predictions from GCMs, and large uncertainties in the model outputs, it is not appropriate to use daily output from GCMs when analysing extreme weather events (Semenov et al., 2010). For each of our 25 sites, we downscaled the climate projections to local-scale scenarios for use in the analysis. Both the baseline and all future climate scenarios were generated using LARS-WG (Semenov et al., 2010), a stochastic weather generator used in many recent European climate change impact and risk assessments (Trnka et al., 2015, 2014; Vanuytrecht et al., 2016), and found to perform well in a range of diverse European climates (Semenov et al., 2013, 2010). LARS-WG downscales the projections from the GCMs and incorporates changes in both the mean climate, climatic variability and extreme events derived from the GCMs (Semenov, 2007), by allowing modification to the statistical distribution of the weather variables. For the baseline climate, site-specific observed daily weather data from 1981 to 2010 was used to estimate site parameters and then LARS-WG was used to generate 300 years of daily weather data with the same statistical characteristics as the observed data. A large number of years (300) were generated to produce daily weather data with probability distributions close to those of the observed baseline climate and accurate reproduction of climatic variability and extreme weather events. For each site, future synthetic daily weather data (300 years) was generated by the LARS-WG weather generator based on changes in distributions of climate variables derived from each GCM and emissions scenario representing the climate in 2050 and 2090. Changes in monthly mean maximum and minimum temperatures and changes in monthly mean precipitation derived from each of the GCMs from the CMIP5 ensemble were incorporated into LARS-WG. Changes in the length of dry/wet spells were not considered due to coarse spatial resolutions of GCMs from CMIP5. For the UK, accounting for changes in the length in dry/wet spells should not affect the main conclusions (Vanuytrecht et al., 2014). In previous studies, LARS-WG demonstrated a good performance to reproduce extreme weather events in diverse climates including the UK (Gitau et al., 2018; Semenov, 2008).

### 2.4 Measuring adverse weather conditions for wheat production

Using the AgriClim software we computed the probability of 7 adverse weather conditions and used a crop simulation model, Sirius, to examine the severity of water, drought and heat stress. We used multiple GCMs, emission scenarios, and two future time periods to contrast a range of possible future climates and provide an indication of the uncertainty in predictions. Table 2 describes the indices used to evaluate changes in adverse weather conditions in the UK under climate change. These indices were developed in previous studies to represent adverse weather conditions during different phenological stages which could lead to crop failure or a significant yield reduction in winter wheat.

**Table 2:** Overview of the adverse weather indices used in this study.

Indicator name		Effect on wheat	Event trigger / Indicator description
1	Frost with no snow	Leaf chlorosis; burning of leaf tips, severe crop damage (Trnka et al., 2010b)	Tmin a ≤ −20 °C for at least 1 day with no or very limited snow cover^b^ (< 1 cm snow)
2	Late frost	After the loss of winter-hardiness leads to leaf chlorosis, floret sterility, damage to lower stem (Gusta and Fowler, 1976; Petr, 1991)	Tmin a is ≤ −2 °C, after mean air temperature is continuously 10 °C (for at least 5 days) and does not drop below 10 °C for more than 2 days in a row
3	Extremely wet early season	Occurrence of diseases, nitrogen leaching, waterlogging and root anoxia (Bartholomeus et al., 2008; Malik et al., 2002)	Soil moisture is at or above field capacity for >60 days from sowing to anthesis. Days with a mean temperature <3 °C are not counted
4	Lodging	Severe reduction of yield and grain quality, through increased harvest losses and exposure to diseases (Berry et al., 2003; Russell and Wilson, 1994)	At least 2 days (from anthesis to 5 days before maturity) with daily precipitation >40 mm, or >20 mm and soil moisture on the previous day at or above field capacity.
5	Grain filling extreme heat	Speeds up development and decreases yield until the growth stops (Nasehzadeh and Ellis, 2017; Savill et al., 2018)	Tmax^c^ > 35 °C for at least 3 days during the period from 5 days after anthesis to maturity
6	Adverse sowing conditions	Restricts the ability to use the appropriate sowing window (Trnka et al., 2014, 2011)	Fewer than 3 days during the sowing window^d^ with the soil moisture in the top layer <90% but >5% and rain on the day is <5 mm and ≤10 mm on the preceding day
7	Adverse harvest conditions	Restricts the ability to harvest at the most appropriate time (Trnka et al., 2014, 2011)	Fewer than 3 days during the harvest window^e^ with soil moisture in the top layer <85% and rain on the given day is <0.5 mm and ≤5 mm on the preceding day
8	Heat stress index (HSI)	Heat stress during the reproductive period causes partial or complete sterility of the florets (Alghabari et al., 2014; Porter and Gawith, 1999)	$\mathrm{HSI}=(1-\frac{Y_{\mathrm{wh}}}{Y_{w}})$ where *Y_wh_* is water and heat limited yield of heat sensitive, drought tolerant, Mercia.
9	Drought stress index (DSI)	Drought stress during the reproductive period causes premature abortion of florets and sterility (Dong et al., 2017; Ma et al., 2017)	$\mathrm{DSI}=(1-\frac{Y_{\mathrm{wd}}}{Y_{w}})$ where *Y_wd_* is water-limited yield of drought sensitive, heat tolerant, Mercia.
10	Water stress index (WSI)	Water stress during the entire growing season causes severe reduction of growth or crop die back (Jamieson et al., 1998)	$\mathrm{WSI}=(1-\frac{Y_{w}}{Y})$ where *Y* is potential yield (not limited by water) of heat and drought tolerant Mercia, *Y_w_* is water-limited yield (rain fed only) of heat and drought tolerant Mercia.

a The Tmin minimum daily temperature was measured 2 m above ground; thus, the actual crop temperature might be even lower.

b The snow cover was estimated using a model validated by Trnka et al. (2010b).

c The Tmax maximum daily temperature was measured 2 m above ground.

d The sowing window is sowing date ±15 days.

e The harvest window is maturity date + 5 days, to maturity + 25 days.

We used the software AgriClim (Trnka et al., 2010a) to compute the probability of a range of adverse weather conditions under the baseline and future climate scenarios, using indices 1–7 (Table 2). These thresholds were used in the European wheat study of Trnka et al. (2014) and determined using a combination of literature and expert judgement. The indicators include the effect of low temperatures. The lethal low temperature according to Porter and Gawith (1999) (−17.2 °C) was modified to incorporate the effect of snow cover. Based on experimental evidence −20 °C was considered a critical low temperature threshold, with no continuous snow cover, causing severe crop damage in winter wheat (Bergjord et al., 2008; Trnka et al., 2010b). Furthermore, following loss of winter-hardiness late frosts can lead to a substantial reduction in yield and based on previous findings a temperature threshold of −2 °C was used, following exposure of wheat to warm temperatures (>10 °C) (Gusta and Fowler, 1976; Petr, 1991). The adverse weather indices also consider the effect of high and heavy precipitation. Extremely wet conditions leading to waterlogging between sowing and anthesis was based upon the number of days with soil moisture at or above the field capacity (Trnka et al., 2014). A high risk of lodging occured with at least 2 days of heavy precipitation or high soil moisture and rainfall between anthesis and maturity (Trnka et al., 2014). The probability of high temperatures during grain filling was measured using the mean lethal maximum temperature (35 °C) identified in Porter and Gawith (1999). The final two indices calculated using AgriClim consider the effect of highly saturated topsoil and precipitation during the sowing or harvesting period and causing highly unsuitable conditions for field operations, making sowing or harvesting impossible (Trnka et al., 2014). The AgriClim software (Trnka et al., 2010a), uses daily inputs of global radiation, maximum and minimum temperature and precipitation to calculate phenological development and the incidence of adverse weather conditions for winter wheat. Sunshine hours were converted to solar radiation using the approach described in Rietveld (1978). The daily reference (ET_r_) evapotranspiration was estimated using the FAO Penman-Monteith method, with wind speed and relative humidity estimated (Allen et al., 1998). Actual evapotranspiration (ET_a_) and soil moisture content were then estimated using the SoilClim water balance model, which accounted at least partly for preferential soil water flow and snow cover (Hlavinka et al., 2011). We used one soil profile across all sites with an available water capacity of 180 mm to focus on the signal from climate projections. The phenological phases were calculated in accordance with the methods described in Olesen et al. (2012), based upon thermal time above a base temperature for the following stages: sowing-emergence-anthesis-maturity. For each scenario, we used the first 50 years of our generated data for initiation of the calculations e.g. the soil moisture or phenological model. The data from this spinoff period were not used in the analyses. The presented results of adverse weather conditions within AgriClim were based on the remaining 250 years of data.

To provide evidence for a comprehensive range of adverse weather conditions, we used the Sirius crop model (Jamieson et al., 1998) to examine the impact of 3 adverse weather conditions on wheat yield, calculating the following indices: heat stress index (HSI) drought stress index (DSI) and water stress index (WSI) (Table 2; indicators 8–10). HSI measures the proportion of yield loss due to the effect of heat stress during the reproductive period, as described in Stratonovitch and Semenov (2015). For the heat sensitive wheat cultivars heat stress occurs at temperatures above 30 °C, during the following 2 periods: 10 days before anthesis to anthesis (meiosis and fertilisation) and 5–12 days after anthesis (beginning of grain filling). DSI measures the proportion of yield loss due to the effect of drought stress during the reproductive period, as described in Senapati et al. (2019b). For the drought sensitive wheat cultivars daily photosynthesis and the rate of leaf senescence depend on the ratio of actual to potential transpiration and drought stress reduces the grain number when the ratio of actual transpiration to potential transpiration falls below 0.9 during the following reproductive period: 10 days before flowering to 5 days after flowering. WSI measures the proportion of yield loss due to water stress during the whole growing season. A water stress factor reduces leaf expansion and accelerates leaf senescence in the water-limited yield, as described in Semenov et al. (2009).

The Sirius crop model is described in detail in Jamieson et al. (1998). To summarise, Sirius is a process based wheat simulation model which uses daily weather data, soil description, and management information (nitrogen applications, irrigation and sowing date) to model phenological development and grain yield, including responses to adverse climatic effects including heat, drought and water stress (Senapati et al., 2019b). Biomass production is calculated from intercepted photosynthetically active radiation and simple partitioning rules are used to calculate grain growth (Jamieson et al., 1998). Sirius has been used frequently in wheat studies and found to perform well under diverse climatic conditions, including the UK and across Europe (Ewert et al., 2002; Jamieson et al., 1998; Martre et al., 2006). We use Sirius version 2018, available from https://sites.google.com/view/sirius-wheat/. Wheat yields were simulated using Sirius for the *Mercia* wheat cultivar grown in the UK, which has been calibrated previously using agronomic experiments in the UK (Lawless et al., 2005; Richter and Semenov, 2005). No nitrogen limitation was considered in this study. A single soil-water profile, Hafren, with an available water capacity of 177 mm was used at all sites. A soil-water profile with a lower available water capacity of 127 mm was also used for comparison, with the results provided in the supplementary material. In the current version of Sirius (2018), there is no direct effect of increased CO_2_ on water-use efficiency (no interaction between CO_2_ and drought), therefore, we are not able to assess this effect on WSI and DSI. However, in previous studies, Sirius was able to simulate well the grain yield of wheat grown under elevated CO_2_ and drought conditions in the FACE experiment at Maricopa (Ewert et al., 2002). We present the mean of each index: HSI, DSI and WSI. In addition, we present the extremes of each indicator using the 95th percentile (HSI95, DSI95 and WSI95); which shows the proportion of yield loss expected to occur on average once every 20 years due to each stress, termed ‘extreme heat stress’, ‘extreme drought stress’ and ‘extreme water stress’ respectively.

For all 25 sites the cultivars used in both models (AgriClim and Sirius) represent winter wheat which is typically sown in the UK between September and November; in this study we used a typical sowing date of 20th October for the baseline and future climate scenarios, consistent with Semenov and Stratonovitch (2015) and Senapati et al. (2019a).

We examine the probability and severity of adverse weather conditions for the baseline and future climate scenarios using the median result from all 16 GCMs, as well as, analysing the range of results across the 16 GCMs to provide an indication of uncertainty. Box plot results are presented for 10 of the 25 sites, providing coverage of the UK wheat growing area (Fig. 1). Maps are produced by interpolating the impact indices from all 25 stations using the inverse distance weighted (IDW) method. IDW is a fast and commonly applied interpolation technique (Lu and Wong, 2008) previously used for interpolation of climatic data and found to perform well at modelling temperature and precipitation (Hadi and Tombul, 2018; Li et al., 2011). Results for the 2090 climate are included in supplementary material.

## 3 Results

### 3.1 Future UK climate

CMIP5 future climate projections generally reflect a trend towards hotter and drier summers and warmer wetter winters for the UK, consistent with the UKCP18 probabilistic climate projections (Lowe et al., 2018). 2050 climate projections from the 16 GCMs analysed predicted an annual average temperature increase from the baseline between 0.4 and 2.5 °C for RCP4.5, and between 0.2 and 3.0 °C for RCP8.5 for all 25 sites in the UK, with greater warming in the summer months (Supplementary Fig. 1). In 2050 sites EH and HX in the far south east of England showed the greatest rise in annual average temperatures (up to 3.0 °C under RCP8.5; Supplementary Table 3). Climate projections for rainfall showed variability throughout the year; the early part of the year (January to April) is predicted to be wetter, whereas summer and early autumn (June to October) is predicted to be drier (Supplementary Fig. 2). The decrease in precipitation during the summer was greatest at sites RR and HX in the South East of England (~20% decrease under RCP8.5), while sites in the North showed the smallest decrease, using the median of GCMs (Supplementary Fig. 2; Supplementary Table 4). Climate projections in 2050 showed a wide range in monthly rainfall predictions across all 16 GCMs, with winter rainfall increasing up to ~40% and decreasing as much as 10% and summer rainfall increasing or decreasing up to ~30% (RCP8.5; Supplementary Table 4).

### 3.2 Advancing anthesis and maturity dates

**Fig. 2:**
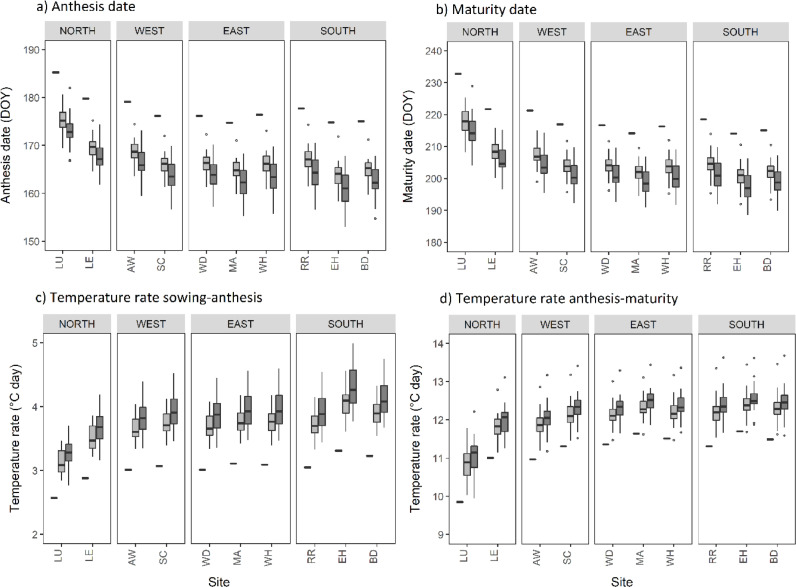
Mean anthesis and maturity dates and values of temperature rate during sowing to anthesis and anthesis to maturity, calculated using AgriClim. Black rectangles indicate the 1981–2010 baseline and box plots indicate the 2050 climate scenarios for RCP4.5 (light grey) and RCP8.5 (dark grey). The calculations consider a medium-ripening cultivar. DOY represents day of year.

Fig. 2 shows the anthesis and maturity dates for winter wheat under baseline and future climate scenarios for 10 sites in the wheat growing area, as simulated using the AgriClim software. Mean anthesis dates, for the 10 sites, were between 10 and 11 days earlier under midrange emissions (RCP4.5) and between 12 and 14 days earlier than the baseline using a high-emissions scenario (RCP8.5). Mean maturity dates were approximately two weeks (13–15 days) earlier under midrange emissions and 16–19 days earlier under high emissions. This advancement is linked to faster crop development under higher temperatures due to a faster rate of thermal time accumulation (Fig. 2). Winter wheat flowers and matures earlier in a warmer climate since the minimal thermal requirement is accumulated faster in both RCP4.5 and RCP8.5 in the mid-21st century, when using a fixed sowing date. This phenological advancement reduces the relative duration of the vegetative and reproduction stages (emergence-anthesis) by up to 2 weeks, whereas the grain filling period (anthesis-maturity) reduces by less than 1 week.

### 3.3 Probability of adverse weather conditions under climate change

**Fig. 3:**
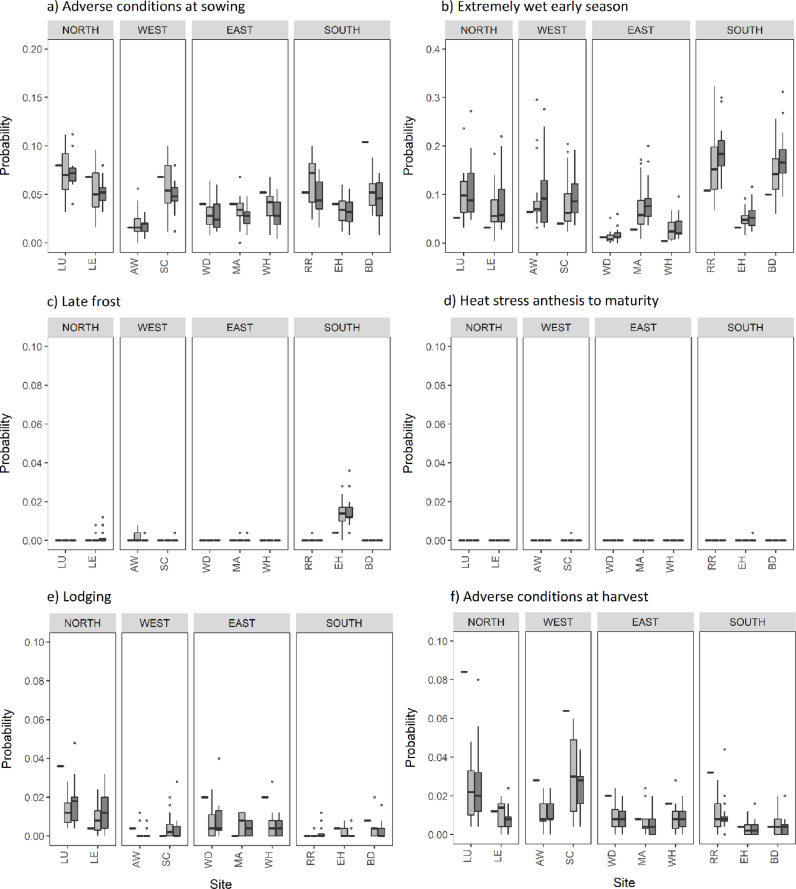
Probability of the occurrence of adverse weather conditions under baseline and 2050 projected climate, calculated using AgriClim. Black rectangles indicate the 1981–2010 baseline and box plots indicate the 2050 climate scenarios for RCP4.5 (light grey) and RCP8.5 (dark grey). The calculations consider a medium-ripening cultivar.

Fig. 3 shows the probability of occurrence of a range of adverse weather conditions, under the baseline and 2050 climate, for RCP4.5 and RCP8.5 emissions scenarios. The box plots for the future climate present the range of results from 16 GCMs in the CMIP5 ensemble.

Sites in the north were consistently the wettest during the sowing period (sowing date ±15 days) under the baseline climate, showing a probability of adverse sowing conditions up to 8%. In contrast, sites in the east are driest, showing a probability less than 5% during the baseline period. The risk of adverse sowing conditions decreased at 8 out of 10 sites under 2050 climate scenarios (and in 2090) as a result of lower soil moisture during the sowing period following a drier summer, as predicted by the CMIP5 ensemble. Sites AW and RR indicated little change or an increase in probability.

**Fig. 4:**
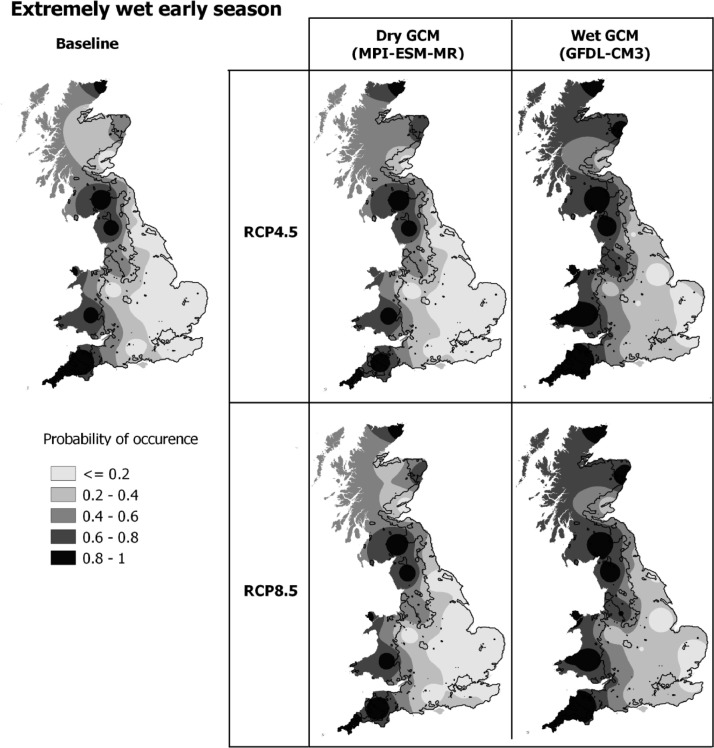
**T**he probability of an extremely wet early season (sowing – anthesis) for the 1981–2010 baseline and 2050 climate using RCP4.5 and RCP8.5 emissions scenarios and dry (MPI-ESM-MR) and wet (GFDL-CM3) GCMs. MPI-ESM-MR is one of the driest models in winter (predicting the largest decrease in rainfall at several sites; supplementary material) and shows a decrease in the probability of an extremely wet early season at a number of UK sites. GFDL-CM3 which is the wettest GCM in winter (shows the largest increase in rainfall; supplementary material) and commonly shows the largest increase in probability of an extremely wet early season.

An extremely wet early season, with possibility of waterlogging between sowing and anthesis, was projected to increase at 9 out of 10 sites under future climates due to increased rainfall and heavy precipitation events in the winter and spring. At sites RR and BD the probability of an extremely wet early season is 10% under the baseline climate, which almost doubles under high emissions in 2050 (and more than doubles in 2090, refer to supplementary material). The maps in Fig. 4 illustrate the probability of an extremely wet early season under baseline and 2050 climate scenarios, with results from all 25 sites interpolated across the UK. The baseline climate in the far west of the country, generally beyond the key wheat growing area, was extremely wet during the early season. For the 2050 climate projections ‘dry’ and ‘wet’ maps use values from the driest and wettest GCMs to illustrate the range of results from the 16 GCMs used in our study. There is a large variation in the probability of an extremely wet early season between GCMs, which is greater than variation in probability of occurrence between emissions scenarios (Figs. 3 and 4). The majority of GCMs showed an increase in the probability of an extremely wet early season, however a smaller number showed a decreased risk under 2050 climate scenarios. The probability of an extremely wet early season using the driest GCM (MPI-ESM-MR) shows there is generally little change in probability compared to the baseline. In contrast, the wettest GCM (GDFL-CM5) shows the probability of waterlogging increases across large areas of the English wheat growing area, as most areas of the country are becoming wetter during the early season.

The risk of a late frost was nil or negligible at 9 sites under the baseline climate and the probability increased slightly (to 1%) at only one site (EH) under 2050 climate scenarios (and in 2090). In the case of a severe frost with no snow cover (figure not presented) the probability was nil during the baseline and future climate scenarios. The probability of heat stress during grain filling (temperatures above 35 °C) was nil or negligible during the baseline and 2050 climate scenarios (and in 2090). The probability of heavy precipitation events between anthesis and maturity, which are a precursor to lodging, was small in the south with a baseline probability less than 1%, which reduced further under 2050 climate scenarios. Results demonstrated variability in the risk of lodging in other regions, the majority of sites showed a decrease under future climate scenarios (in 2050 and 2090), however the probability of lodging increased slightly at sites LE, SC and MA (up to 1%). The probability of adverse conditions at harvest was predicted to decrease under 2050 climate scenarios (and in 2090) across all regions, driven by hotter and drier summers reducing soil moisture at harvest.

### 3.4 Severity of heat, drought and water stress under climate change

**Fig. 5:**
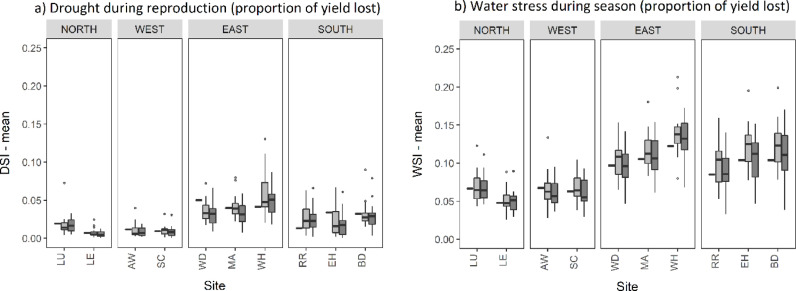
Mean drought stress index (DSI) and water stress index (WSI). Black rectangles indicate the 1981–2010 baseline and box plots indicate the 2050 climate scenarios for RCP4.5 (light grey) and RCP8.5 (dark grey).

Fig. 5 shows the mean proportion of yield loss as a result of drought stress during reproduction (DSI) and water stress during the season (WSI), under baseline and 2050 climate scenarios, simulated using Sirius. Mean heat stress around anthesis (HSI) was nil or negligible under baseline and future climate scenarios.

Mean DSI was highest in the east of the UK with an average of 0.04–0.05 under the baseline climate, representing a 4–5% yield loss as a result of drought stress during reproductive development. In contrast, the north and west regions experienced the lowest drought stress, with DSI between 0.01 and 0.02 under the baseline climate. Most sites showed a decrease in mean drought stress during the reproductive period by 2050, with exception of sites WH and RR, which showed an increase.

Mean WSI ranged from 0.05 to 0.12 under the baseline climate, representing a 5–12% yield loss as a result of water stress during the entire growing season, with the highest water stress in the south and east of the UK (9–12% yield loss). Under midrange emissions in 2050 the mean proportion of yield loss due to water stress increased by up to 25% in the south and east regions (to 10–14%). Under high emissions in 2050, however, sites in these regions show a smaller increase in WSI (less than 10%) in comparison to the baseline climate. The north and west regions experienced the least water stress with WSI less than 0.07 under the baseline climate and small change or a reduction in WSI under 2050 climate scenarios.

### 3.5 Extremes of heat, drought and water stress under the future climate

We used the 95th percentile of heat (HSI95) and drought stress (DSI95) during reproductive development and water stress (WSI95) over the entire wheat growing season to analyse extremes, termed as ‘extreme heat stress’, ‘extreme drought stress’ and ‘extreme water stress’ respectively. HSI95, DSI95 and WSI95 indicate the corresponding proportion of yield losses expected to occur on average once every 20 years. The proportion of yield loss due to extreme heat stress during the reproductive period (HSI95) was nil or negligible under baseline, 2050 and 2090 climate scenarios.

**Fig. 6:**
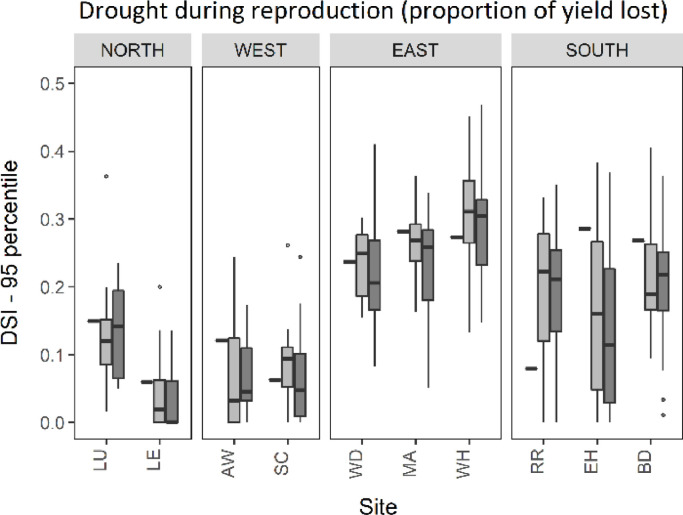
95-percentile drought stress index (DSI95). Black rectangles indicate the 1981–2010 baseline and box plots indicate the 2050 climate scenarios for RCP4.5 (light grey) and RCP8.5 (dark grey).

**Fig. 7:**
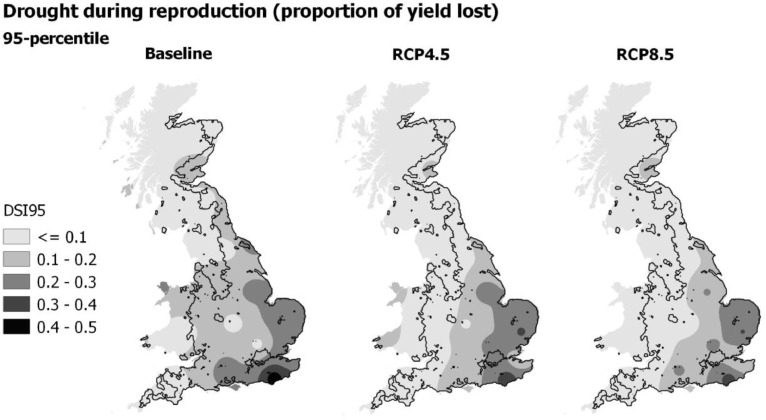
95-percentile of drought stress index (DSI95) for the 1981–2010 baseline and median 2050 climate using RCP4.5 and RCP8.5 emissions scenarios.

Fig. 6 shows extreme drought stress (DSI95) under baseline and 2050 climate for RCP4.5 and RCP8.5 emissions scenarios. Fig. 7 illustrates spatial patterns in DSI95, with results from the 25 sites interpolated across the UK, using the median of GCMs. DSI95 was consistently highest at sites in the east under the baseline climate, between 0.24 and 0.27, representing 24–27% yield loss as a result of extreme drought during reproduction. DSI95 was also high in the south under the baseline climate, with high spatial variability. The highest extreme drought stress during reproductive development occurred in the far south east of England, as indicated by the darkest area in Fig. 7. In contrast, other areas in Southern England experienced the lowest extreme drought stress, with the minimum at site RR (DSI95 <0.1). Overall, the north and west regions had the lowest extreme drought stress during reproduction, with less than 15% yield loss under the baseline climate. Consistent with the mean DSI, most sites showed a decrease in extreme drought stress during reproduction (DSI95) by 2050 (and by 2090, refer to supplementary material). Our projections show a reduction in DSI95 across most regions of the UK under midrange emissions with a further reduction under high emissions (Fig. 7). DSI95 was predicted to reduce by almost half by 2050 at site EH in South East England, reducing from 29% yield loss under the baseline climate to 16% under RCP4.5, and further to 11% yield loss under RCP8.5. However, the box plot at site EH shows a large range, with results from all 16 GCMs showing greater uncertainty compared to other sites. In contrast, small areas in the UK projected an increased drought stress around anthesis by 2050. At site RR, DSI95 more than doubled from 8% under the baseline period to 22% and 21% under midrange and high emissions respectively, with little difference between emission scenarios.

**Fig. 8:**
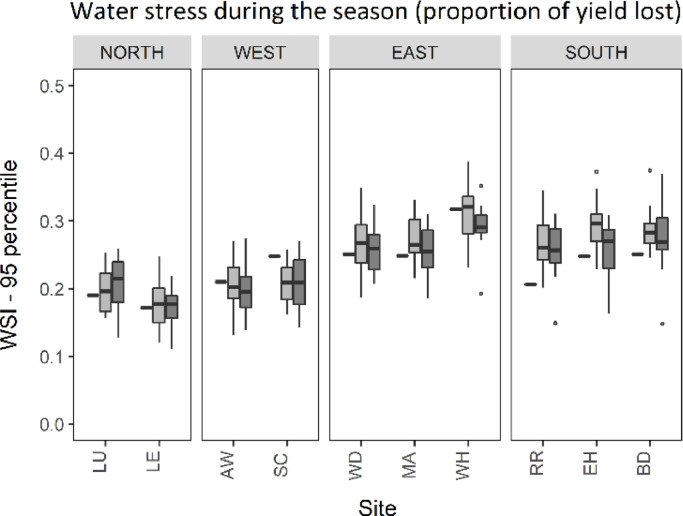
95-percentile water stress index (WSI95). Black rectangles indicate the 1981–2010 baseline and box plots indicate the 2050 climate scenarios for RCP4.5 (light grey) and RCP8.5 (dark grey).

**Fig. 9:**
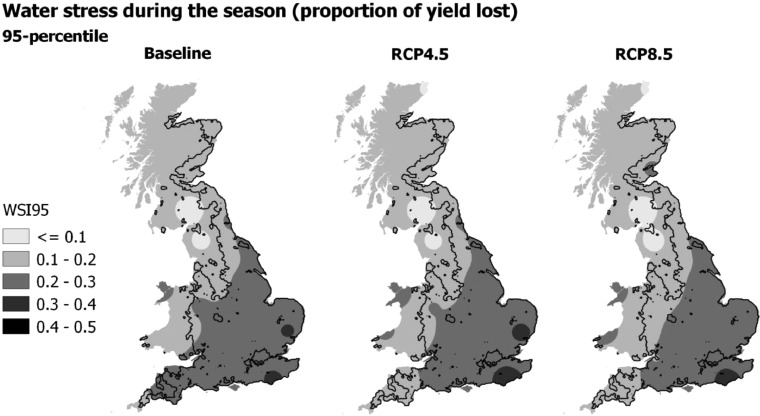
95-percentile of water stress index (WSI95) for the 1981–2010 baseline and median 2050 climate using RCP4.5 and RCP8.5 emissions scenarios.

Across most of the wheat growing area in England extreme water stress during the growing season (WSI95) ranged between 0.20 and 0.30, representing 20 and 30% possible yield losses under the baseline climate (Figs. 8 and 9). WSI95 was highest in the south and east of the country under the baseline climate. Extreme water stress was lowest (WSI95<0.20) across the north of the UK. However less spatial variation was found between sites for extreme water stress than extreme drought stress. At most sites WSI95 increased slightly (less than 0.05) between the baseline and future climate, therefore very little change in WSI95 was shown, with exception of the far west wheat growing area which showed a decrease in extreme water stress during the entire growing season (Fig. 9). Extreme water stress was predicted to be greater under midrange emissions (RCP4.5) than high emissions (RCP8.5) at most sites in the south and east, with a greater increase in rainfall during the winter and spring under RCP8.5. In 2090, a reduction in extreme water stress was projected at most sites across the UK due to further increase in the winter and spring rainfall under RCP8.5.

Uncertainty in simulating the impacts of extreme drought (DSI95) and extreme water stress (WSI95) is highlighted by the range of projections of different GCMs (Figs. 6 and 8) with some showing increases in risk and some showing decreases by 2050. This range is consistent with the variation in predicted monthly rainfall between the baseline and 2050 climate; with summer rainfall increasing or decreasing up to ~30% across all 16 GCMs and winter rainfall increasing up to ~40% and decreasing as much as 10% (RCP8.5). Greater variation is shown for extreme drought events during the reproductive period (DSI95) in comparison to water stress during the growing season (WSI95), highlighting particular uncertainty in GCM model predictions for simulating extreme drought during the reproductive period.

## 4 Discussion

Wheat is sensitive to various climatic stresses throughout the growing season. Overall, future climates in the UK are expected to remain favourable for wheat production under a midrange (RCP4.5) and a high emissions scenario (RCP8.5), with most adverse weather indicators reducing in frequency or magnitude during the 21st century. Drier summers are expected to reduce the probability of overly wet conditions during sowing and around the harvest period, which can restrict the ability to sow or harvest at the most appropriate time (Trnka et al., 2014). The risk of lodging which can lead to yield losses and a reduction in quality (Berry et al., 2003; Gobin, 2018; Russell and Wilson, 1994; Trnka et al., 2015), is also expected to decrease with fewer heavy rainfall events prior to and at maturity.

The risk of severe winter frosts, which can lead to severe crop damage including leaf chlorosis, following exposure to temperatures below −20 °C with no snow cover (Trnka et al., 2014), would most likely be zero under future climate, as temperatures will not fall this low. Late frosts, which occur after the loss of winter hardiness at temperatures below −2 °C, cause leaf chlorosis, floret sterility during anthesis and damage the lower stem, leading to medium to severe yield loss (Gusta and Fowler, 1976; Petr, 1991). The risk of a late frost seems negligible at all sites throughout the UK wheat growing area.

High temperatures and heat stress around anthesis could induce sterility and considerable yield loss in wheat, with a critical temperature during reproduction around 30 °C (Alghabari et al., 2014; Semenov et al., 2014; Porter and Gawith, 1999). Previous research found a small risk of heat stress around anthesis (>30 °C) at one site in South East England (RR) under mid-century climate scenarios (Semenov et al., 2014; Semenov and Shewry, 2011) and we found consistent results throughout the UK, with negligible yield losses due to heat stress during reproduction under both baseline and future climate scenarios. The probability of heat stress during grain filling (> 35 °C), which can reduce grain size and quality (Nasehzadeh and Ellis, 2017; Savill et al., 2018) was also negligible.

The probability of an extremely wet early season, driven by increased rainfall between sowing and anthesis, would increase under future climates across most of the UK wheat growing area. This can cause waterlogging, root anoxia, and fertilizer leaching (Trnka et al., 2014). Furthermore, wetter winters, coupled with the predicted warmer temperatures and fewer frosts may increase the prevalence of pests and disease such as *Zymoseptoria tritici* (Fones and Gurr, 2015; Pietravalle et al., 2007)*.* The impact of pests and disease on wheat yield was not analysed in this study. Other landscape characteristics, including soil type and slope of the land, can influence the probability of waterlogging which were not analysed.

An increase in precipitation during the winter and early spring may reduce the impacts of hotter and drier summers under future climates in the UK. Prolonged water stress during the growing season reduces leaf expansion, accelerates leaf senescence and subsequently reduces yield (Jamieson et al., 1998). Despite a decrease in rainfall between May and October, water stress is predicted to decrease across England and Wales for two reasons also indicated by Semenov (2009): Firstly, additional winter rainfall would be stored in the soil, depending on the available water capacity, and made available to the crop during the dry period. Secondly, winter wheat would mature earlier in a warmer climate, therefore reducing exposure to the hotter drier period towards the end of the crop growth. Senapati et al. (2019b) also found a low probability of severe drought during reproduction in mid-century climate scenarios, at site RR in South East England. Our results show the proportion of yield loss due to drought stress during reproduction is generally higher in the south and east of the UK, which receives less rainfall than the north and west. Drought stress is however spatially and temporally diverse, showing variation between sites within the same region, and variation in future climate predictions. Yield loss due to drought stress is likely to decrease across most of the UK under future climates, with exceptions of two sites (RR and WH) which show an increase. We used one soil profile across all sites with an available water capacity of 177 mm (for drought and water stress calculations in Sirius) to focus on the signal from climate projections. However, soil depth and soil type, as well as, other landscape characteristics can influence the frequency and severity of adverse weather conditions, including short-term drought and prolonged water stress. Using a light soil, with an available water capacity of 127 mm, the relative yield losses due to drought and water stress were found to be substantially greater than when using a medium soil (refer to supplementary material). Relative yield losses due to water stress in the future climate are generally expected to be similar to the baseline or increase slightly across the south and east of the UK. However, water stress for sites in the west is expected to decrease. Climate signals indicate vulnerability to water stress will not increase considerably in the UK, as may be expected in other parts of the world. However, our results show that the impact of a changing climate on water and drought stress is spatially specific and likely to depend on local environmental conditions, including soil characteristics.

Different studies predicted the risk of adverse weather conditions, and subsequent crop failure under climate change across a large region of Europe (Trnka et al., 2015, 2011). The risk of heat stress and drought was projected to increase across Southern Europe, particularly around the Mediterranean (Olesen et al., 2011; Trnka et al., 2014). Furthermore, regions of Northern Europe (Scandinavia) are expected to suffer more from frost stress due to lower temperatures in the future climate (Trnka et al., 2014). However, the present study shows that the temperate climate of the UK is expected to be suitable for growing wheat in the future. The UK already dedicates a large proportion of agricultural land to wheat production and it may be difficult to expand outside of the current growing area with the west experiencing very wet conditions in the early part of the season. Efforts are therefore required to increase wheat production for future food security in the current growing region, through greater intensification or enabling wheat to cope better with region and season-specific climatic threats. The severity of adverse weather conditions will depend on cultivar characteristics. Our results highlight the importance of research focusing on early season waterlogging, which mostly occurs in the western growing regions but is expected to increase throughout the UK under future climates. Prolonged water stress will not increase considerably in the UK, but greater tolerance to water stress would help to increase overall yields by minimising ongoing yield losses for wheat grown in the south and east of the UK.

In the current study, we used future projections from 16 GCMs from CMIP5 to analyse adverse weather conditions for UK wheat production. The multi-model median provides an estimate of future conditions compared to the baseline climate, however the distribution of projections also provides important information about uncertainty. Predictions for the 2050 climate show a wide range in monthly rainfall predictions, which lead to uncertainty in the results of different adverse weather indices, for example: adverse conditions at sowing, wet early season and drought and water stress, as indicated by the wide range in results. At many sites the minimum values for these adverse weather conditions show a decrease in risk, whereas the maximum values show an increase. There is also generally a larger difference between the GCMs (minimum and maximum values) than between emission scenarios, highlighting the importance of using a range of models in the analysis of extreme and adverse weather conditions.

Our results highlight the importance of looking at a range of sites across the UK to provide results at a smaller spatial scale, in order to make inferences about the weather related risks for UK wheat production, and guide local adaptation or growing area expansion. Weather across the UK shows large spatial variation under the baseline and future climate, thus climate risk assessment relevant to wheat production needs to be analysed at a local scale, particularly when considering the risk of drought stress.

Underpredicting inter-annual variability is a well-known issue with weather generators including LARS-WG. However, this should not affect the calculation of adverse weather conditions analysed because the indices are based on extreme weather formulated using daily values, often during a short period of the crop development. It has been shown that LARS-WG reproduces extreme weather events well (Gitau et al., 2018; Semenov, 2008).

## 5 Conclusion

The UK climate is expected to remain favourable for wheat production, with most adverse weather indicators reducing in magnitude during the 21st century. Hotter and drier summers, and warmer wetter winters are expected to lead to improved sowing and harvest conditions, along with a reduced risk of lodging. The risk of late frosts and probability of heat stress during reproductive and grain filling periods would likely remain low in the future across the UK. The rainfall patterns appear more influential for wheat production in the UK. The probability of a wetter winter and spring, which generally cause issues with waterlogging, leaching and root anoxia in the western wheat growing regions, are expected to increase throughout the UK in the future. The severity of drought stress during the reproductive period is generally lower in the future climate, however there are localised differences across the wheat growing area and accordingly it is important to examine drought episodes at a small spatial scale so that adaptation can be targeted efficiently. Prolonged water stress does not seem to increase considerably in the UK, as may be expected in other parts of Europe and the world.

Climate predictions from the CMIP5 ensemble show a wide range in projections for monthly precipitation, and relative changes from the baseline climate. Based on adverse weather indices, our study shows GCMs revealed uncertainty in the adverse weather conditions, including waterlogging and yield losses due to drought and water stresses. This variation in adverse weather indicators due to GCMs is generally greater than the variation between RCP emissions scenarios. Accordingly, GCM ensembles should be used in the assessment of adverse weather conditions for crop production to indicate the full range of possible impacts, which a limited number of GCMs may not provide.

In the present study, we analysed the frequency and magnitude of a range of adverse weather conditions, which have been identified within the literature as resulting in a significant yield reduction. However, with existing process-based crop models, including Sirius, it is not possible to quantify the impact of all adverse weather conditions analysed in this study on wheat yields, for example waterlogging and lodging. In order to examine these impacts the adverse weather conditions and abiotic stresses simulated in crop models could be expanded. Similarly, the subsequent impact on farm income was not well known. In order to understand the full impact of adverse weather conditions on crop production, and in turn farm income, these aspects should be considered in future research to develop farm resilience and address future food insecurity, in a changing climate.

## Declaration of Competing Interest

The authors declare that they have no known competing financial interests or personal relationships that could have appeared to influence the work reported in this paper.

## Appendix Supplementary materials

Image, application 1
