# Supplementary material for: Adverse weather conditions for UK wheat production under climate change
Source: Agric For Meteorol. 2020 Mar 15;282-283:107862. doi: 10.1016/j.agrformet.2019.107862 (PMC7001962; doi:10.1016/j.agrformet.2019.107862)
Supplement: Supplementary file 1 [file mmc1.docx]

**Supplementary Material**

**Adverse weather conditions for UK wheat production under climate change**

**Future UK climate**
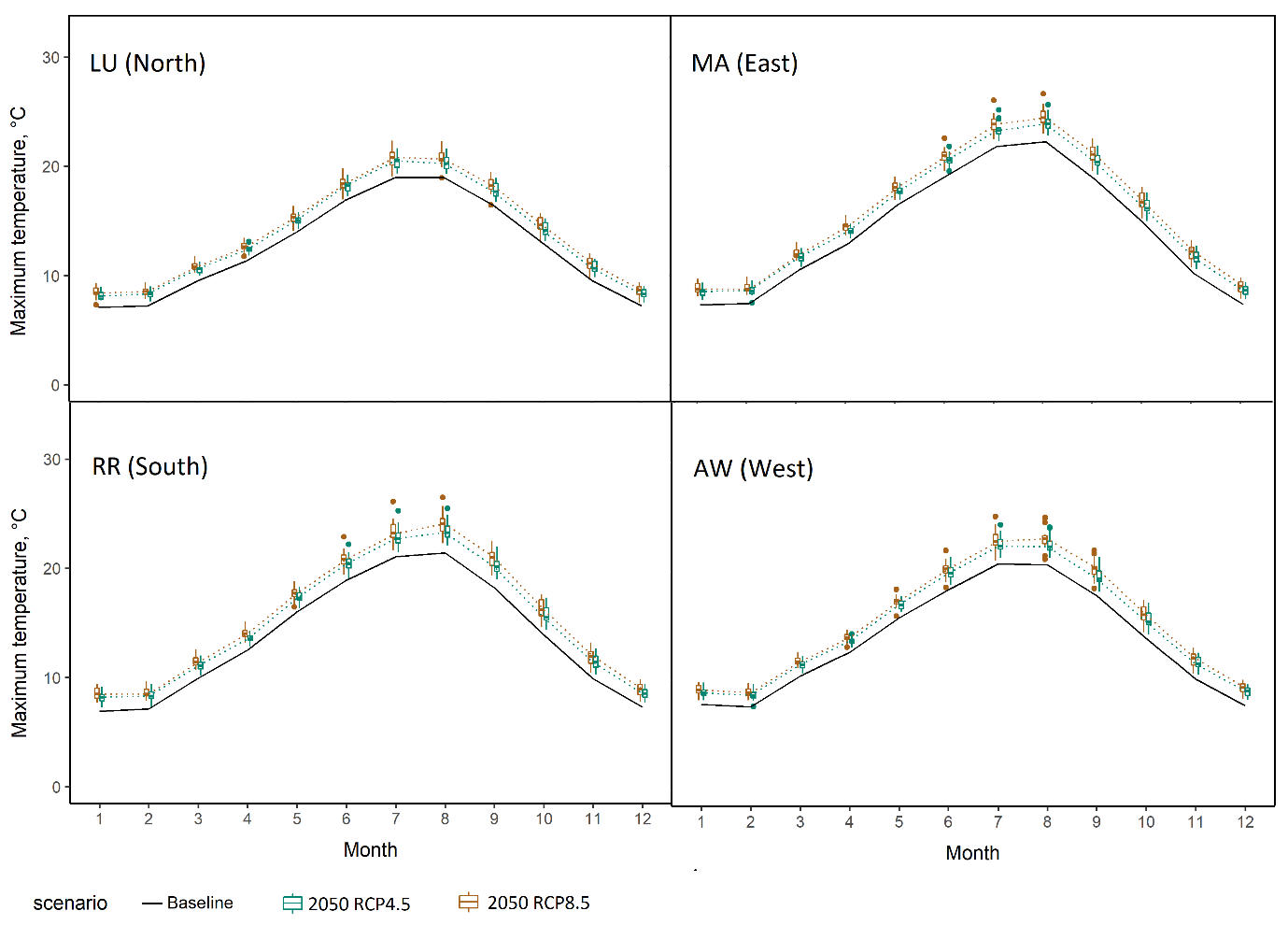


Supplementary Figure 1 – Mean maximum monthly temperature for the 1981-2010 baseline (solid line) and box plots for the 2050 climate scenarios (RCP4.5 and RCP8.5). At four sites across the UK wheat growing area.


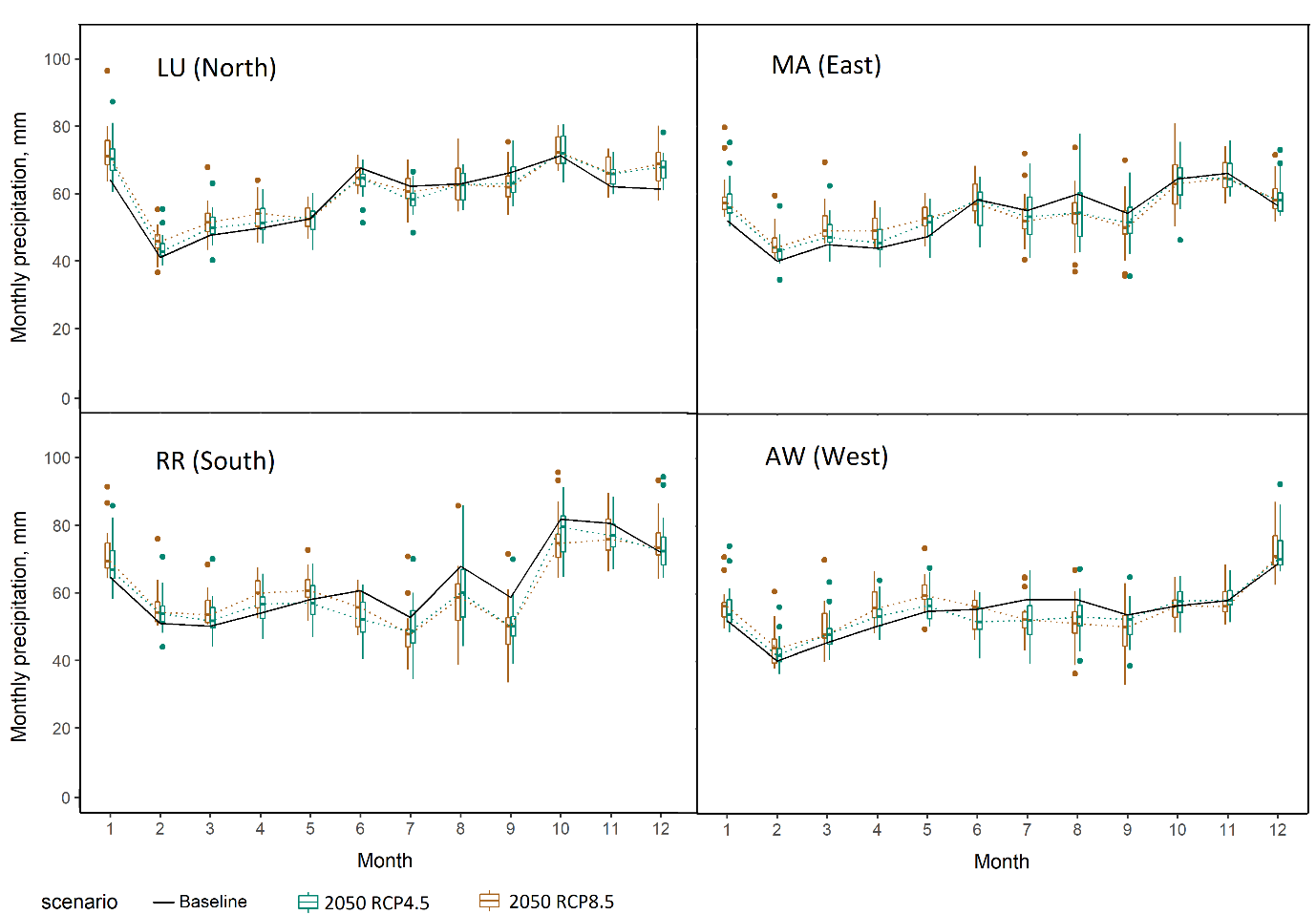


Supplementary Figure 2 - Mean monthly precipitation for the 1981-2010 baseline (solid line) and box plots for the 2050 climate scenarios (RCP4.5 and RCP8.5). At four sites across the UK wheat growing area.

**Frequency and severity of adverse weather conditions in 2090**

The probability of an extremely wet early season for wheat production is projected to increase by the 2050 (as discussed in the main text). Supplementary Figure 3 provides the probability of an extremely wet early season at all 25 sites and all climate scenarios, illustrating an increasing trend across time and emissions scenarios. In 2090 the probability of an extremely wet early season is expected to increase more than two-fold at 10 sites across the wheat growing area under high emissions (RCP8.5), including sites RR, MA and WH which are located within a key wheat growing region of the South East.


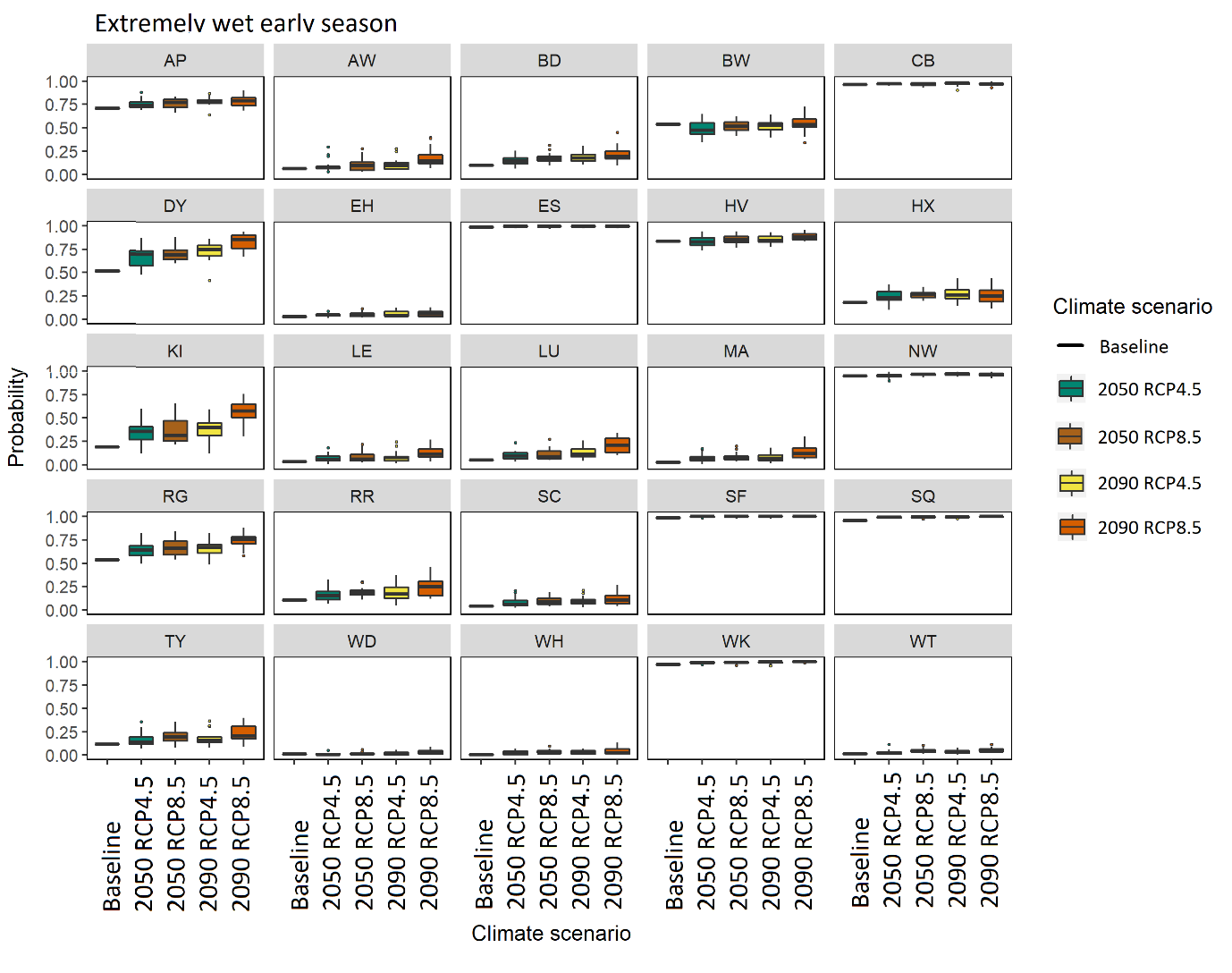


Supplementary Figure 3 - Probability of the occurrence of extremely wet early season under baseline and projected climate. Black rectangles indicate the 1981-2010 baseline. Box plots indicate the 2050 climate scenarios for RCP4.5 and RCP8.5 and 2090 climate scenarios for RCP4.5 and RCP8.5, from left to right. The calculations consider a medium-ripening cultivar.

Supplementary Figure 4 and Supplementary Figure 5 provide the 95-percentile of drought stress index (DSI95) and water stress index (WSI95), respectively, at all 25 sites and all climate scenarios.

In 2090 most sites project a lower DSI95 under both RCP4.5 and RCP8.5 in comparison to the baseline period. In contrast site RR is unique in projecting an increase in DSI95 by 2100 under both emission scenarios. In 2090, using the median of GCMs, most sites show little change or a lower WSI95 under RCP8.5 in comparison to the baseline climate. HSI95 (heat stress index) continues to be negligible or very low in 2090.


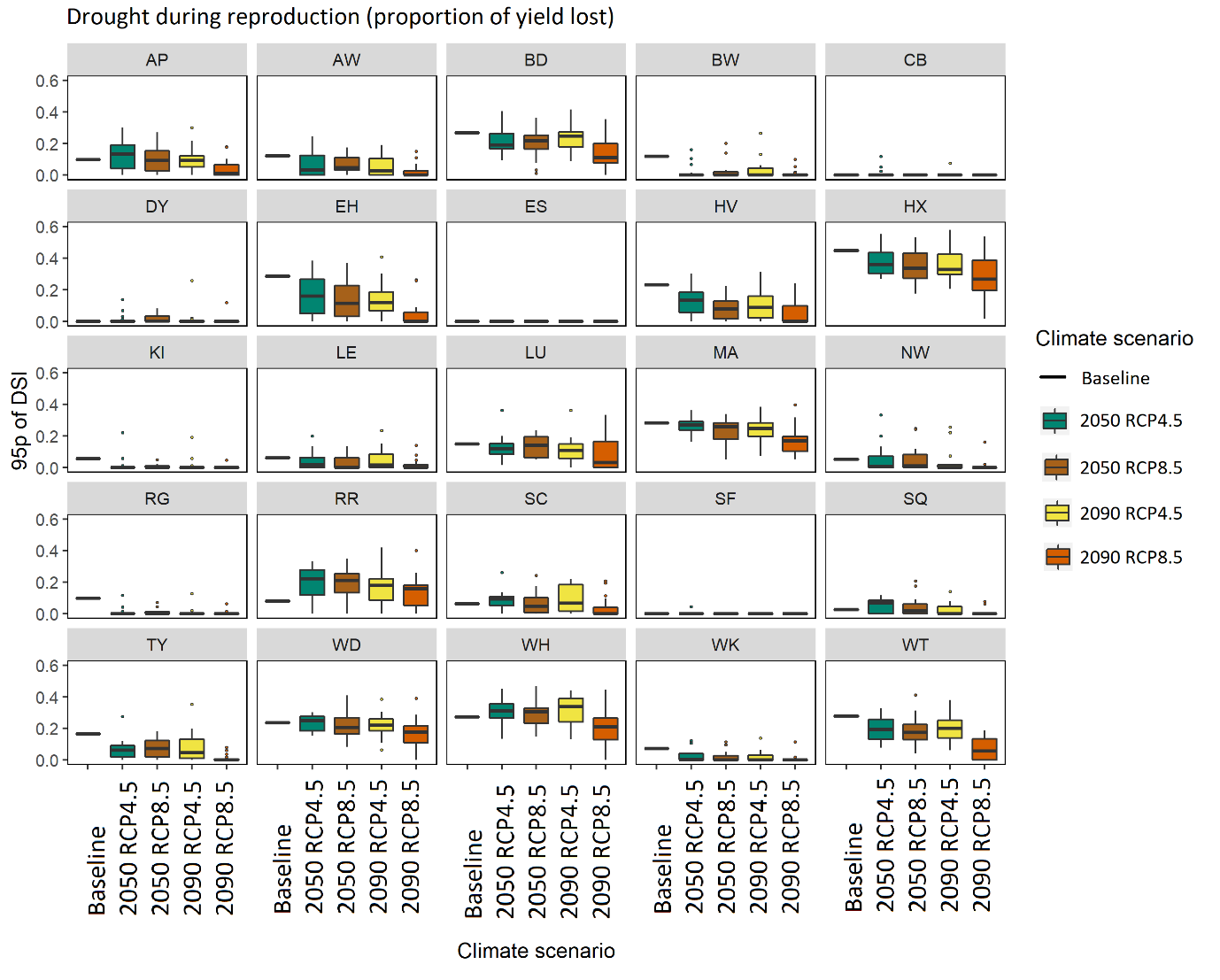


Supplementary Figure 4 - 95-percentile of drought stress index (DSI95) Black rectangles indicate the 1981-2010 baseline. Box plots indicate the 2050 climate scenarios for RCP4.5 and RCP8.5 and 2090 climate scenarios for RCP4.5 and RCP8.5, from left to right.


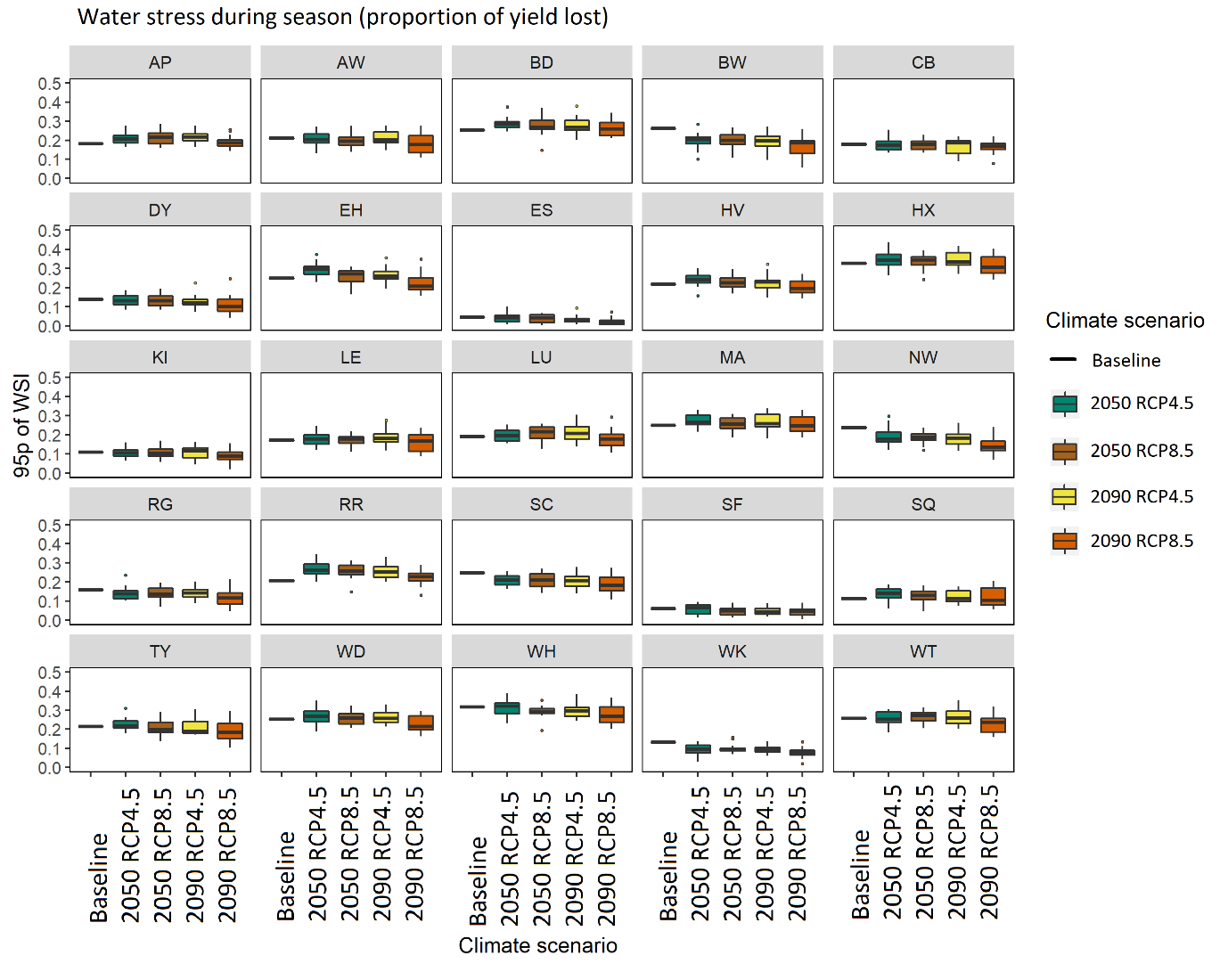


**Supplementary Figure 5 - 95-percentile of water stress index (WSI95)** Black rectangles indicate the 1981-2010 baseline. Box plots indicate the 2050 climate scenarios for RCP4.5 and RCP8.5 and 2090 climate scenarios for RCP4.5 and RCP8.5, from left to right.

**Range of projections from the CMIP5 ensemble**

For most sites CSIRO-MK36 (GCM model no. 3) is the driest model in summer, with the largest reduction in rainfall between the baseline and 2050 climate, as illustrated by Supplementary Figure 6, however this is cooler than other models. For most sites GFDL-CM3 (GCM model no. 5) is the hottest model in the CMIP5 ensemble in the summer, which is also consistently very dry across UK sites. INM-CM4 (GCM model no. 9) is frequently the coldest model in summer and MIROC-ESM (GCM model no. 11) commonly the wettest.

For most sites GFDL-CM3 is the wettest model in winter, with the largest increase in precipitation between the baseline and 2050s (RCP8.5) as illustrated by Supplementary Figure 6. For most sites MIROC-ESM the hottest model in the CMIP5 ensemble for winter. CSIRO-MK36 is the coldest model in winter. A number of GCMs predict no change or a small decrease in winter precipitation, however there is not one model which is consistently the driest in winter across all sites in the UK. MPI-ESM-MR (GCM no. 12) is the driest for site RR and a number of other sites.


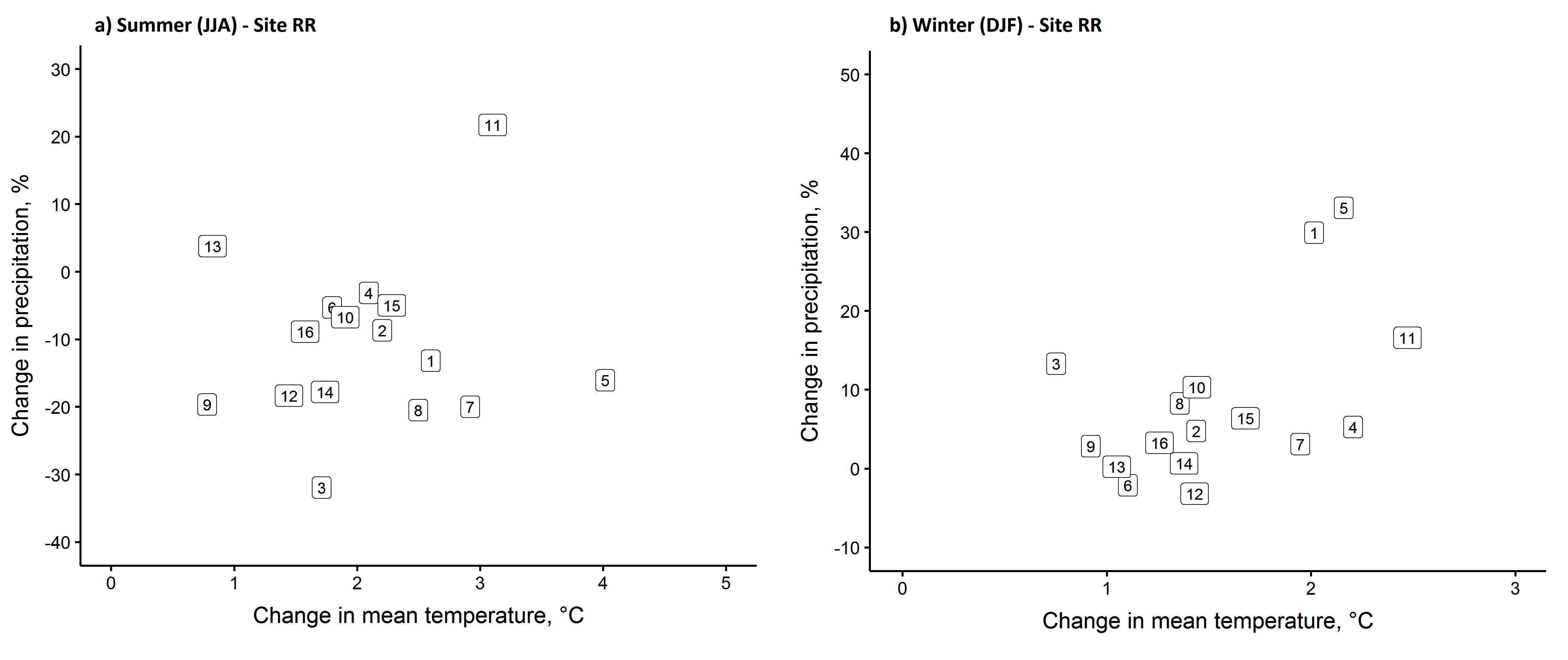


Supplementary Figure 6 – Absolute changes in mean temperature and relative changes in mean precipitation at site RR (Rothamsted Research) during a) the summer months (JJA) and b) the winter months (DJF), between baseline (1981-2010) and 2050 climate, under RCP8.5 for 16 GCMs from the CMIP5 ensemble. Model numbers correspond with Supplementary Table 2.

**Drought and water stress for light soils**

We consider the effect of soil available water capacity (AWC) on extreme drought stress (DSI95) and extreme water stress (WSI95) (Supplementary Figure 7). The AWC used in these calculations represents a medium and light soil, with the majority of wheat in England and Wales grown on soils with an AWC 95-215 mm (Clarke, 2017; Hallett et al., 1996)

**
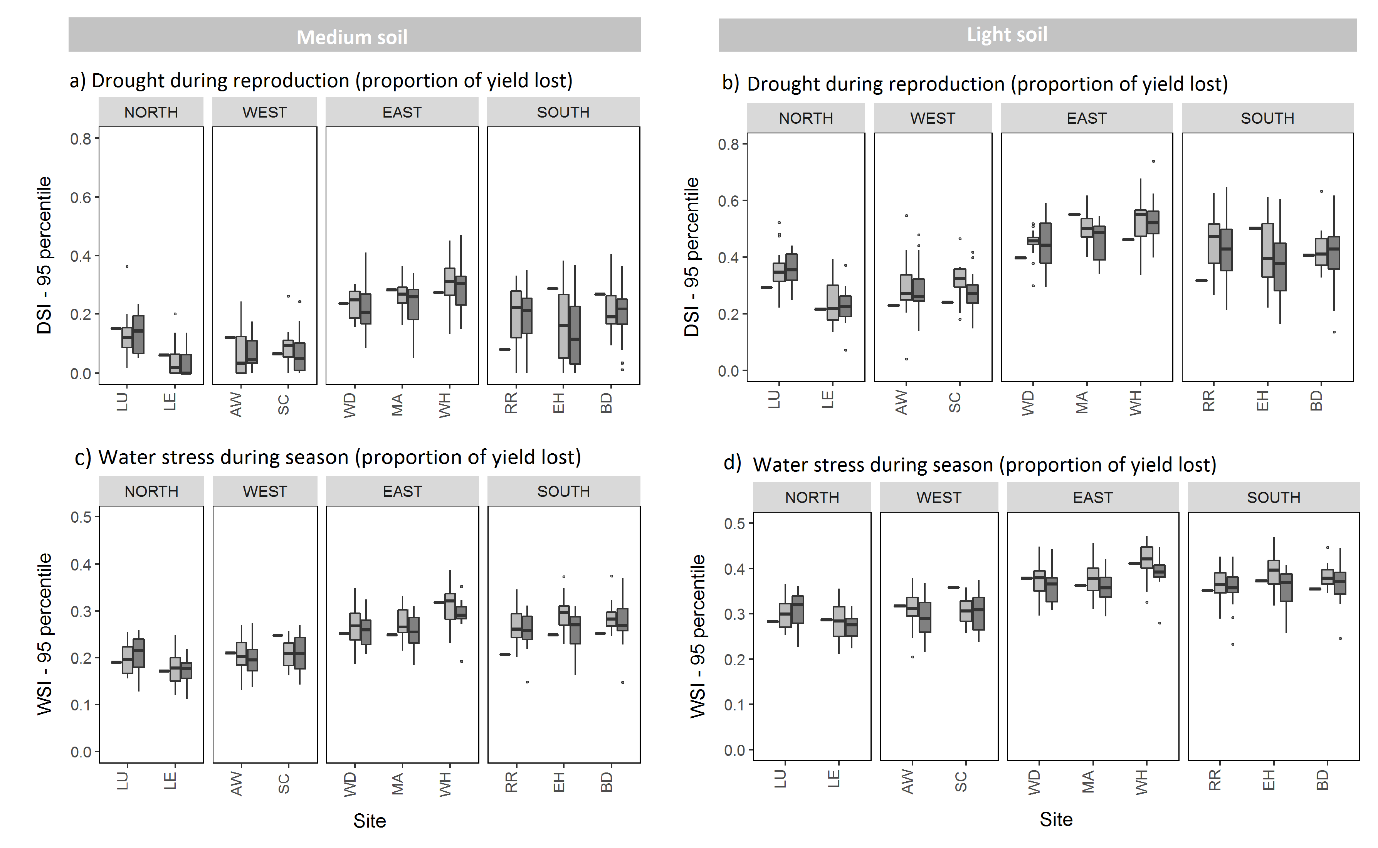
Supplementary Figure 7 – 95-percentile of drought stress index (DSI95) (a, b) and water stress index (WSI95) (c, d)** The first column (a, c) shows results for the medium soil with AWC of 177 mm (as used in Figures 5-9 and Supplementary Figures 4-5), and the second column (b, d) shows results for light soil with AWC of 127 mm. Black rectangles indicate the 1981-2010 baseline and box plots indicate the 2050 climate scenarios for RCP4.5 (light grey) and RCP8.5 (dark grey).

Supplementary Table 1 - Characteristics of the 25 UK sites for the 1981-2010 baseline conditions

The mean dates of the phenological stages represent the AgriClim model estimates for early-, medium- and late-ripening cultivars. (The sowing, anthesis and maturity dates are expressed as the day of the year – DOY from January 1st).

| **Site** | **Acronym** | **Lat** | **Long** | **Alt** | **Mean precip.** | **Mean temp, °C** | | **Sowing DOY** |
| --- | --- | --- | --- | --- | --- | --- | --- | --- |
|  |  |  |  |  | **Annual, mm** | **Jan, min** | **Jul, max** |  |
| Wick | WK | 58.45 | -3.09 | 36 | 793 | 1.7 | 15.7 | 293 |
| Kinloss | KI | 57.65 | -3.56 | 5 | 688 | 1.3 | 18.5 | 293 |
| Dyce | DY | 57.21 | -2.20 | 65 | 812 | 1.2 | 18.1 | 293 |
| Leuchars | LU | 56.38 | -2.86 | 10 | 709 | 0.6 | 19.0 | 293 |
| Eskdalemuir | ES | 55.31 | -3.21 | 242 | 1747 | -0.1 | 17.8 | 293 |
| Tynemouth | TY | 55.02 | -1.42 | 33 | 631 | 2.4 | 18.2 | 293 |
| Shap Fell | SF | 54.50 | -2.68 | 255 | 1753 | 0.5 | 18.0 | 293 |
| Whitby | WT | 54.48 | -0.60 | 41 | 561 | 2.1 | 18.6 | 293 |
| Leeming | LE | 54.30 | -1.53 | 32 | 643 | 1.2 | 20.7 | 293 |
| Ringway | RG | 53.36 | -2.28 | 33 | 805 | 1.7 | 20.4 | 293 |
| Holyhead Valley | HV | 53.25 | -4.54 | 10 | 848 | 4.0 | 18.3 | 293 |
| Waddington | WD | 53.18 | -0.52 | 68 | 602 | 1.5 | 20.9 | 293 |
| Shawbury | AW | 52.79 | -2.66 | 72 | 652 | 1.0 | 20.7 | 293 |
| Marham | MA | 52.65 | 0.57 | 21 | 644 | 0.9 | 21.8 | 293 |
| Church Lawford | SC | 52.36 | -1.33 | 107 | 676 | 1.3 | 21.4 | 293 |
| Wattisham | WH | 52.12 | 0.96 | 89 | 626 | 1.3 | 21.3 | 293 |
| Aberporth | AP | 52.14 | -4.57 | 133 | 857 | 3.2 | 17.9 | 293 |
| Sennybridge | SQ | 52.06 | -3.61 | 307 | 1375 | 0.9 | 18.0 | 293 |
| Rothamsted | RR | 51.80 | -0.35 | 128 | 751 | 1.2 | 21.0 | 293 |
| Bristol | BW | 51.45 | -2.60 | 42 | 855 | 3.5 | 21.7 | 293 |
| East Hamsted | EH | 51.38 | 0.78 | 75 | 680 | 1.6 | 22.1 | 293 |
| Boscombe Down | BD | 51.16 | -1.75 | 126 | 751 | 1.6 | 21.7 | 293 |
| North Wyke | NW | 50.77 | -3.90 | 177 | 1065 | 2.6 | 19.7 | 293 |
| Herstmonceux | HX | 50.89 | 0.32 | 52 | 787 | 2.4 | 20.7 | 293 |
| Camborne | CB | 50.22 | -5.33 | 87 | 1037 | 4.7 | 18.3 | 293 |

** In the case of the late cultivar at Eskdalemuir and Wick, maturity was not reached.*

Supplementary Table 2 - Global climate models from the CMIP5 ensemble used in the LARS-WG weather generator. The scenarios are based on RCP4.5 and RCP8.5 for the periods 1981-2010 (baseline) and 2041-2060 and 2081-2100.

| **Model no.** | **Research centre** | **Country** | **Global climate model** | **Grid resolution** | **Reference** |
| --- | --- | --- | --- | --- | --- |
| 1 | The Centre for Australian Weather and Climate Research | Australia | ACCESS1-3 | 1.25° x 1.88° | (Collier and Uhe, 2012) |
| 2 | Canadian Centre for Climate Modelling and Analysis | Canada | CanESM2 | 2.77° x 2.81° | (Chylek et al., 2011) |
| 3 | Australia's Commonwealth Scientific and Industrial Research Organisation | Australia | CSIRO-MK36 | 1.85° x 1.88° | (Jeffrey et al., 2013) |
| 4 | EC-EARTH consortium | Europe | EC-EARTH | 1.125° x 1.125° | (Hazeleger et al., 2012) |
| 5 | Geophysical Fluid Dynamics Laboratory | USA | GFDL-CM3 | 2° x 2.5° | (Griffies et al., 2011) |
| 6 | Goddard Institute for Space Studies National | USA | GISS-E2-R-CC | 2.00° x 2.50° | (Chandler et al., 2013) |
| 7 | UK Meteorological Office | UK | HadGEM2-ES | 1.25° x 1.88° | (Collins et al., 2011; Jones et al., 2011) |
| 8 | Institute Pierre Simon Laplace | France | IPSL-CM5A-MR | 1.27° x 2.50° | (Dufresne et al., 2013) |
| 9 | Institute for Numerical Mathematics | Russia | INM-CM4 | 1.50° x 20° | (Volodin et al., 2013; Yurova and Volodin, 2011) |
| 10 | University of Tokyo, National Institute for Envir. Studies, Japan Agency for Marine-Earth Science & Technology | Japan | MIROC5 | 1.39° x 1.41° | (Mochizuki et al., 2012; Tatebe et al., 2012; Watanabe et al., 2011) |
| 11 |  |  | MIROC-ESM | 2.77° x 2.81° | (Watanabe et al., 2011) |
| 12 | Max-Planck Institute for Meteorology | Germany | MPI-ESM-MR | 1.85° x 1.88° | (Brovkin et al., 2013; Schmidt et al., 2013) |
| 13 | Meteorological Research Institute | Japan | MRI-CGCM3 | 1.11° x 1.13° | (Tsujino et al., 2011) |
| 14 | National Centre for Atmospheric Research | USA | NCAR-CCSM4 | 0.94° x 1.25° | (Jahn and Holland, 2013; Meehl et al., 2013) |
| 15 |  |  | NCAR-CESM1-CAM5 | 0.94° x 1.25° | (Jahn and Holland, 2013) |
| 16 | Norwegian Climate Centre | Norway | NorESM1-M | 1.90° x 2.50° | (Bentsen et al., 2013; Iversen et al., 2013) |

**Supplementary Table 3 –** **Annual mean temperature for the 1981-2010 baseline and change in annual mean temperature under future climate scenarios (RCP4.5 and RCP8.5).**

Minimum and maximum values show the smallest and largest change in temperature (respectively) across all 16 GCMs, from the baseline.

|  | **Annual mean temp, °C** | **Change in annual mean temp, from baseline, °C** | | | |
| --- | --- | --- | --- | --- | --- |
| **Site** | **Baseline** | **RCP4.5** | | **RCP8.5** | |
|  |  | **Min GCM** | **Max GCM** | **Min GCM** | **Max GCM** |
| AP | 9.9 | 0.5 | 2.1 | 0.5 | 2.5 |
| AW | 9.6 | 0.6 | 2.2 | 0.6 | 2.7 |
| BD | 10.1 | 0.6 | 2.3 | 0.8 | 2.8 |
| BW | 11.2 | 0.6 | 2.3 | 0.7 | 2.9 |
| CB | 10.9 | 0.4 | 1.9 | 0.4 | 2.4 |
| DY | 8.6 | 0.5 | 1.9 | 0.3 | 2.4 |
| EH | 10.2 | 0.5 | 2.5 | 0.8 | 3.0 |
| ES | 7.6 | 0.6 | 2.0 | 0.4 | 2.4 |
| HV | 10.5 | 0.5 | 2.0 | 0.5 | 2.5 |
| HX | 10.6 | 0.4 | 2.5 | 0.8 | 3.0 |
| KI | 8.9 | 0.5 | 1.9 | 0.3 | 2.3 |
| LE | 9.5 | 0.6 | 2.1 | 0.5 | 2.6 |
| LU | 8.8 | 0.6 | 2.0 | 0.4 | 2.4 |
| MA | 10.0 | 0.6 | 2.2 | 0.7 | 2.7 |
| NW | 10.0 | 0.5 | 2.2 | 0.5 | 2.7 |
| RG | 10.0 | 0.6 | 2.2 | 0.6 | 2.7 |
| RR | 9.8 | 0.6 | 2.4 | 0.8 | 2.9 |
| SC | 9.9 | 0.6 | 2.2 | 0.7 | 2.7 |
| SF | 8.0 | 0.6 | 2.0 | 0.5 | 2.5 |
| SQ | 8.4 | 0.6 | 2.1 | 0.6 | 2.6 |
| TY | 9.4 | 0.6 | 2.1 | 0.5 | 2.6 |
| WD | 9.9 | 0.6 | 2.2 | 0.7 | 2.7 |
| WH | 10.0 | 0.6 | 2.3 | 0.7 | 2.8 |
| WK | 8.0 | 0.4 | 1.8 | 0.2 | 2.2 |
| WT | 9.4 | 0.7 | 2.2 | 0.5 | 2.7 |

**Supplementary Table 4 – Mean precipitation for the 1981-2010 baseline, for summer (JJA) and winter (DJF) and change in precipitation under future climate scenarios (RCP4.5 and RCP8.5).**

Minimum and maximum values show the smallest and largest change in precipitation (respectively) across all 16 GCMs, from the baseline.

|  | **Mean precip., summer, mm** | **Change in summer precip., from baseline, %** | | | | **Mean precip., winter, mm** | **Change in winter precip., from baseline, %** | | | |
| --- | --- | --- | --- | --- | --- | --- | --- | --- | --- | --- |
| **Site** | **Baseline** | **RCP4.5** | | **RCP8.5** | | **Baseline** | **RCP4.5** | | **RCP8.5** | |
|  |  | **Min GCM** | **Max GCM** | **Min GCM** | **Max GCM** |  | **Min GCM** | **Max GCM** | **Min GCM** | **Max GCM** |
| AP | 64 | -25 | 12 | -26 | 12 | 77 | -4 | 35 | -6 | 38 |
| AW | 57 | -28 | 13 | -26 | 10 | 54 | -5 | 34 | -5 | 31 |
| BD | 52 | -26 | 16 | -25 | 22 | 73 | -8 | 26 | -3 | 34 |
| BW | 63 | -25 | 19 | -26 | 20 | 84 | -10 | 28 | -6 | 31 |
| CB | 66 | -21 | 13 | -23 | 13 | 112 | -5 | 31 | -4 | 35 |
| DY | 63 | -11 | 9 | -11 | 10 | 66 | -3 | 32 | -7 | 37 |
| EH | 47 | -25 | 27 | -25 | 30 | 61 | -8 | 28 | -4 | 32 |
| ES | 129 | -21 | 10 | -19 | 7 | 178 | -5 | 36 | -3 | 32 |
| HV | 62 | -18 | 14 | -20 | 14 | 74 | -5 | 39 | -5 | 41 |
| HX | 56 | -28 | 23 | -28 | 22 | 76 | -8 | 27 | -5 | 30 |
| KI | 63 | -9 | 5 | -15 | 10 | 54 | -6 | 24 | -10 | 31 |
| LE | 58 | -20 | 16 | -20 | 18 | 51 | -4 | 34 | -6 | 41 |
| LU | 64 | -13 | 6 | -11 | 8 | 55 | -1 | 33 | -4 | 39 |
| MA | 58 | -23 | 20 | -26 | 23 | 50 | -7 | 34 | 1 | 38 |
| NW | 63 | -23 | 15 | -27 | 10 | 118 | -6 | 27 | -5 | 29 |
| RG | 65 | -25 | 16 | -24 | 18 | 71 | -5 | 38 | -6 | 39 |
| RR | 60 | -31 | 20 | -32 | 22 | 62 | -8 | 28 | -3 | 33 |
| SC | 64 | -27 | 18 | -24 | 18 | 49 | -1 | 34 | -2 | 38 |
| SF | 99 | -21 | 12 | -19 | 10 | 204 | -9 | 32 | -8 | 30 |
| SQ | 89 | -19 | 14 | -23 | 18 | 136 | -2 | 35 | -3 | 36 |
| TY | 53 | -18 | 10 | -20 | 12 | 49 | -5 | 37 | -8 | 39 |
| WD | 60 | -24 | 12 | -28 | 17 | 45 | -4 | 30 | -5 | 36 |
| WH | 59 | -24 | 24 | -26 | 25 | 45 | -4 | 37 | 4 | 43 |
| WK | 60 | -13 | 8 | -17 | 11 | 66 | -2 | 30 | -8 | 32 |
| WT | 46 | -21 | 10 | -21 | 14 | 48 | -1 | 40 | -3 | 42 |

**References**

Bentsen, M., Bethke, I., Debernard, J.B., Iversen, T., Kirkevåg, A., Seland, Ø., Drange, H., Roelandt, C., Seierstad, I.A., Hoose, C., Kristjánsson, J.E., 2013. The Norwegian Earth System Model, NorESM1-M – Part 1: Description and basic evaluation of the physical climate. Geosci. Model Dev. 6, 687–720. https://doi.org/10.5194/gmd-6-687-2013

Brovkin, V., Boysen, L., Raddatz, T., Gayler, V., Loew, A., Claussen, M., 2013. Evaluation of vegetation cover and land-surface albedo in MPI-ESM CMIP5 simulations. J. Adv. Model. Earth Syst. 5, 48–57. https://doi.org/10.1029/2012MS000169

Chandler, M.A., Sohl, L.E., Jonas, J.A., Dowsett, H.J., Kelley, M., 2013. Simulations of the mid-Pliocene Warm Period using two versions of the NASA/GISS ModelE2-R Coupled Model. Geosci. Model Dev. 6, 517–531. https://doi.org/10.5194/gmd-6-517-2013

Chylek, P., Li, J., Dubey, M.K., Wang, M., Lesins, G., 2011. Observed and model simulated 20th century Arctic temperature variability: Canadian Earth System Model CanESM2. Atmos. Chem. Phys. Discuss. 11, 22893–22907. https://doi.org/10.5194/acpd-11-22893-2011

Clarke, D., 2017. Assessing the impacts of drought on UK wheat production. Cranfield University.

Collier, M., Uhe, P., 2012. CMIP5 datasets from the ACCESS1.0 and ACCESS1.3 coupled climate models.

Collins, W.J., Bellouin, N., Doutriaux-Boucher, M., Gedney, N., Halloran, P., Hinton, T., Hughes, J., Jones, C.D., Joshi, M., Liddicoat, S., Martin, G., Connor, F., Rae, J., Senior, C., Sitch, S., Totterdell, I., Wiltshire, A., Woodward, S., 2011. Development and evaluation of an Earth-System model – HadGEM2. Geosci. Model Dev. 4, 1051–1075. https://doi.org/10.5194/gmd-4-1051-2011

Dufresne, J.-L., Foujols, M.-A., Denvil, S., Caubel, A., Marti, O., Aumont, O., Balkanski, Y., Bekki, S., Bellenger, H., Benshila, R., Bony, S., Bopp, L., Braconnot, P., Brockmann, P., Cadule, P., Cheruy, F., Codron, F., Cozic, A., Cugnet, D., de Noblet, N., Duvel, J.-P., Ethé, C., Fairhead, L., Fichefet, T., Flavoni, S., Friedlingstein, P., Grandpeix, J.-Y., Guez, L., Guilyardi, E., Hauglustaine, D., Hourdin, F., Idelkadi, A., Ghattas, J., Joussaume, S., Kageyama, M., Krinner, G., Labetoulle, S., Lahellec, A., Lefebvre, M.-P., Lefevre, F., Levy, C., Li, Z.X., Lloyd, J., Lott, F., Madec, G., Mancip, M., Marchand, M., Masson, S., Meurdesoif, Y., Mignot, J., Musat, I., Parouty, S., Polcher, J., Rio, C., Schulz, M., Swingedouw, D., Szopa, S., Talandier, C., Terray, P., Viovy, N., Vuichard, N., 2013. Climate change projections using the IPSL-CM5 Earth System Model: from CMIP3 to CMIP5. Clim. Dyn. 40, 2123–2165. https://doi.org/10.1007/s00382-012-1636-1

Griffies, S.M., Winton, M., Donner, L.J., Horowitz, L.W., Downes, S.M., Farneti, R., Gnanadesikan, A., Hurlin, W.J., Lee, H.-C., Liang, Z., Palter, J.B., Samuels, B.L., Wittenberg, A.T., Wyman, B.L., Yin, J., Zadeh, N., 2011. The GFDL CM3 Coupled Climate Model: Characteristics of the Ocean and Sea Ice Simulations. J. Clim. 24, 3520–3544. https://doi.org/10.1175/2011JCLI3964.1

Hallett, S.H., Jones, R.J.A., Keay, C.A., 1996. Environmental information systems developments for planning sustainable land use. Int. J. Geogr. Inf. Syst. 10, 47–64. https://doi.org/10.1080/02693799608902066

Hazeleger, W., Wang, X., Severijns, C., Ştefǎnescu, S., Bintanja, R., Sterl, A., Wyser, K., Semmler, T., Yang, S., van den Hurk, B., van Noije, T., van der Linden, E., van der Wiel, K., 2012. EC-Earth V2.2: Description and validation of a new seamless earth system prediction model. Clim. Dyn. 39, 2611–2629. https://doi.org/10.1007/s00382-011-1228-5

Iversen, T., Bentsen, M., Bethke, I., Debernard, J.B., Kirkevåg, A., Seland, Ø., Drange, H., Kristjansson, J.E., Medhaug, I., Sand, M., Seierstad, I.A., 2013. The Norwegian Earth System Model, NorESM1-M – Part 2: Climate response and scenario projections. Geosci. Model Dev. 6, 389–415. https://doi.org/10.5194/gmd-6-389-2013

Jahn, A., Holland, M.M., 2013. Implications of Arctic sea ice changes for North Atlantic deep convection and the meridional overturning circulation in CCSM4-CMIP5 simulations. Geophys. Res. Lett. 40, 1206–1211. https://doi.org/10.1002/grl.50183

Jeffrey, S., Rotstayn, L., Collier, M., Dravitzki, S., Hamalainen, C., Moeseneder, C., Wong, K., Skytus, J., 2013. Australia’s CMIP5 submission using the CSIRO-Mk3.6 model. Aust. Meteorol. Oceanogr. J. 63, 1–14. https://doi.org/10.22499/2.6301.001

Jones, C.D., Hughes, J.K., Bellouin, N., Hardiman, S.C., Jones, G.S., Knight, J., Liddicoat, S., O&amp;apos;Connor, F.M., Andres, R.J., Bell, C., Boo, K.-O., Bozzo, A., Butchart, N., Cadule, P., Corbin, K.D., Doutriaux-Boucher, M., Friedlingstein, P., Gornall, J., Gray, L., Halloran, P.R., Hurtt, G., Ingram, W.J., Lamarque, J.-F., Law, R.M., Meinshausen, M., Osprey, S., Palin, E.J., Parsons Chini, L., Raddatz, T., Sanderson, M.G., Sellar, A.A., Schurer, A., Valdes, P., Wood, N., Woodward, S., Yoshioka, M., Zerroukat, M., 2011. The HadGEM2-ES implementation of CMIP5 centennial simulations. Geosci. Model Dev. 4, 543–570. https://doi.org/10.5194/gmd-4-543-2011

Meehl, G.A., Washington, W.M., Arblaster, J.M., Hu, A., Teng, H., Kay, J.E., Gettelman, A., Lawrence, D.M., Sanderson, B.M., Strand, W.G., 2013. Climate change projections in CESM1(CAM5) compared to CCSM4. J. Clim. 26, 6287–6308. https://doi.org/10.1175/JCLI-D-12-00572.1

Mochizuki, T., Chikamoto, Y., Kimoto, M., Ishii, M., Tatebe, H., Komuro, Y., Sakamoto, T.T., Watanabe, M., Mori, M., 2012. Decadal Prediction Using a Recent Series of MIROC Global Climate Models. J. Meteorol. Soc. Japan 90A, 373–383. https://doi.org/10.2151/jmsj.2012-A22

Schmidt, H., Rast, S., Bunzel, F., Esch, M., Giorgetta, M., Kinne, S., Krismer, T., Stenchikov, G., Timmreck, C., Tomassini, L., Walz, M., 2013. Response of the middle atmosphere to anthropogenic and natural forcings in the CMIP5 simulations with the Max Planck Institute Earth system model. J. Adv. Model. Earth Syst. 5, 98–116. https://doi.org/10.1002/jame.20014

Tatebe, H., Ishii, M., Mochizuki, T., Chikamoto, Y., Sakamoto, T.T., Komuro, Y., Mori, M., Yasunaka, S., Watanabe, M., Ogochi, K., Suzuki, T., Nishimura, T., Kimoto, M., 2012. The Initialization of the MIROC Climate Models with Hydrographic Data Assimilation for Decadal Prediction. J. Meteorol. Soc. Japan 90A, 275–294. https://doi.org/10.2151/jmsj.2012-A14

Tsujino, H., Hirabara, M., Nakano, H., Yasuda, T., Motoi, T., Yamanaka, G., 2011. Simulating present climate of the global ocean–ice system using the Meteorological Research Institute Community Ocean Model (MRI.COM): simulation characteristics and variability in the Pacific sector. J. Oceanogr. 67, 449–479. https://doi.org/10.1007/s10872-011-0050-3

Volodin, E.M., Diansky, N.A., Gusev, A. V., 2013. Simulation and Prediction of Climate Changes in the 19th to 21st Centuries with the Institute of Numerical Mathematics, Russian Academy of Sciences, Model of the Earth’s Climate System. Известия Российской академии наук. Физика атмосферы и океана 49, 379–400. https://doi.org/10.7868/S000235151304010X

Watanabe, S., Hajima, T., Sudo, K., Nagashima, T., Takemura, T., Okajima, H., Nozawa, T., Kawase, H., Abe, M., Yokohata, T., Ise, T., Sato, H., Kato, E., Takata, K., Emori, S., Kawamiya, M., 2011. MIROC-ESM 2010: model description and basic results of CMIP5-20c3m experiments. Geosci. Model Dev. 4, 845–872. https://doi.org/10.5194/gmd-4-845-2011

Yurova, A.Y., Volodin, E.M., 2011. Coupled simulation of climate and vegetation dynamics. Izv. Atmos. Ocean. Phys. 47, 531–539. https://doi.org/10.1134/S0001433811050124
